# Robust Nitrogen-Doped Microporous Carbon via Crown Ether-Functionalized Benzoxazine-Linked Porous Organic Polymers for Enhanced CO_2_ Adsorption and Supercapacitor Applications

**DOI:** 10.1021/acsami.4c05645

**Published:** 2024-07-22

**Authors:** Mohamed Gamal Mohamed, Bo-Xuan Su, Shiao-Wei Kuo

**Affiliations:** † Department of Materials and Optoelectronic Science, Center of Crystal Research, National Sun Yat-Sen University, Kaohsiung 804, Taiwan; ‡ Chemistry Department, Faculty of Science, Assiut University, Assiut 71516, Egypt; § Department of Medicinal and Applied Chemistry, Kaohsiung Medical University, Kaohsiung 807, Taiwan

**Keywords:** benzoxazine, crown ether, porous organic polymers, microporous carbon, CO_2_ uptake, energy storage

## Abstract

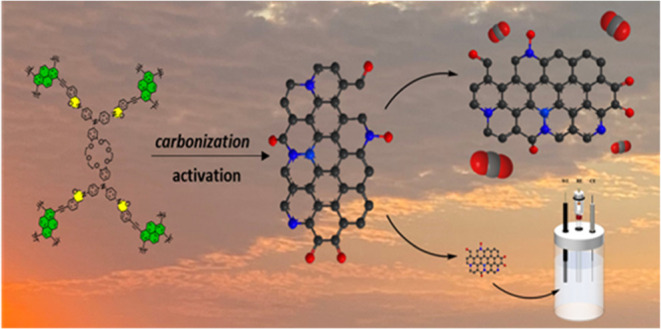

Nitrogen-doped carbon materials, characterized by abundant microporous and nitrogen functionalities, exhibit significant potential for carbon dioxide capture and supercapacitors. In this study, a class of porous organic polymer (POP) were successfully synthesized by linking Cr-TPA-4BZ-Br_4_ and tetraethynylpyrene (Py-T). The model benzoxazine monomers of Cr-TPA-4BZ and Cr-TPA-4BZ-Br_4_ were synthesized using the traditional three-step method [involving CH=N formation, reduction by NaBH_4_, and Mannich condensation]. Subsequently, the Sonogashira coupling reaction connected the Cr-TPA-4BZ-Br_4_ and Py-T monomers, forming Cr-TPA-4BZ-Py-POP. The successful synthesis of Cr-TPA-4BZ-Br_4_ and Cr-TPA-4BZ-Py-POP was confirmed through various analytical techniques. After verifying the successful synthesis of Cr-TPA-4BZ-Py-POP, carbonization and KOH activation procedures were conducted. These crucial steps led to the formation of poly(Cr-TPA-4BZ-Py-POP)-800, a carbon material with a structure akin to graphite. In practical applications, poly(Cr-TPA-4BZ-Py-POP)-800 exhibited a noteworthy CO_2_ adsorption capacity of 4.4 mmol/g, along with specific capacitance values of 397.2 and 159.2 F g^–1^ at 0.5 A g^–1^ (measured in a three-electrode cell) and 1 A g^–1^ (measured in a symmetric coin cell), respectively. These exceptional dual capabilities stem from the optimal ratio of heteroatom doping. The outstanding performance of poly(Cr-TPA-4BZ-Py-POP)-800 microporous carbon holds significant promise for addressing contemporary energy and environmental challenges, making substantial contributions to both sectors.

## Introduction

Globally, from 1901 to 2018, there has been a rise in sea levels (SLR) ranging between 15 and 25 cm, averaging 1–2 mm per year. However, the rate of sea level rise has accelerated to 4.62 mm/year during the decade from 2013 to 2022.^1^ The primary driver of this phenomenon is human-induced climate change. The contribution to the rise in sea level was as follows: temperate glaciers melting accounted for 21%, Greenland ice caps melting contributed 15%, Antarctic ice caps melting contributed 8%, and thermal expansion of saltwater contributed 42%.^1,2^ Addressing the pressing issues of global warming caused by greenhouse gas emissions, energy substitution, and efficient energy utilization is imperative given the substantial energy demands of humankind.^3^

Nitrogen-doped carbon materials have garnered widespread attention in materials science, chemistry, and nanotechnology.^4−8^ These materials effectively enhance the conductivity, catalytic activity, and adsorption performance of carbon materials.^4−8^ The introduction of nitrogen also allows for adjustment of the chemical reactivity of carbon materials. N-doped carbon materials find diverse applications in supercapacitors, fuel cells, chemical catalysts, and carbon dioxide adsorption.^9,10^ The N-doping process primarily involves synthesizing chemical groups containing nitrogen atoms onto the precursor and then subjecting it to carbonization, utilizing substances such as phenolic resin, biomass derivatives polyacrylonitrile, polypyrrole, biomass, polyaniline, and so on.^11−14^

Hyper-cross-linked polymers (HPP), covalent triazine frameworks (CTFs), covalent organic frameworks (COFs), and conjugated microporous polymers (CMPs) represent four instances of porous organic polymers (POPs) characterized by substantial specific surface areas and well-defined pores, explored for a broad range of applications.^15−17^ These applications encompass chemical sensing, energy storage, hydrogen evolution, gas capture, and separation, as well as photocatalysis.^15−17^ Schiff base and coupling reactions [including Suzuki, Yamamoto, and Sonogashira] are among the chemical reactions often used in POP synthesis. The purpose of these reactions is usually to add covalent bonds to the polymer structure, such as boroxine, imine, and triazine units.^18−24^ The optoelectronic and thermal characteristics of POPs can be customized for potential applications by employing diverse synthesis techniques and incorporating various building blocks.^18−24^ In the realms of chemical sensing, photocatalysis, CO_2_ adsorption, batteries, supercapacitors, and H_2_ generation, these POPs have exhibited promising performance.^25,26^ Some groups have successfully modified POPs by introducing additional functional groups, including amine/amide, quinolone, oxazole, and thiazole, through solid-state chemical transformation processes.^27−31^ For example, the imine-linked covalent organic framework (COF) can undergo a transition into thiocarbamate and carbamate-linked COFs with increased *S*_BET_.^32^ Utilizing reduction and Mannich reaction, certain researchers have employed benzoxazine-linked COF, exhibiting an *S*_BET_ exceeding 650 m^2^/g.^33^ Our group prepared 3D TPM-BZ-Py POP with *S*_BET_ around 185 m^2^/g.^34^

Polybenzoxazines (PBZs), a unique category of heterocyclic polymers, are formed by polymerizing benzoxazine (BZ) monomers using ring-opening technology.^35−37^ Through the thermal curing of oxazine units without the need for a curing agent or catalyst, BZs, a distinctive category of thermosetting materials, can form inter- and intramolecular hydrogen bonds. These materials find widespread applications in coatings, low-dielectric materials, and aerospace.^38−40^ BZ monomers can typically be generated by employing aromatic phenols, aromatic or aliphatic amines, and CH_2_O or aromatic aldehydes in the Mannich process.^38−40^ Furthermore, there has been a proposal for synthesizing POPs linked by benzoxazine with substantial surface areas. For instance, Tan et al. synthesized BZ-linked POPs with *S*_BET_ up to 230 m^2^/g using a one-step Mannich reaction involving triphenol and CH_2_O.^41^ Recently, we widened the range of BZ-linked POPs with superior *S*_BET_ by creating various derivatives of building monomers. This was achieved through the utilization of Sonogashira-Hagihara coupling to create various brominated BZ compounds with unique building blocks that have been ethynyl functionalized.^41−43^

Through the distribution of micropore sizes, microporous carbons (MCs) could reduce ion-transport resistance and diffusion distance within the pores, resulting in enhanced electrochemical capacitance.^44^ Nitrogen-doped MCs contribute to improving the electronic conductivity and surface wettability. Additionally, owing to the nitrogen functionality embedded in the carbon framework, these materials facilitate reversible pseudocapacitance through Faradaic electrochemical interactions occurring at the interfaces of the electrolyte and electrodes.^45^ Consequently, MCs are widely considered as excellent electrode materials for supercapacitors, demonstrating outstanding performance.^45^ PBZs also exhibit great potential for high-performance nitrogen-doped MCs due to their robust heat stability, high char yield, robust heat stability, and less shrinkage.^45,46^ Moreover, the nitrogen (N) content in these materials could be modified by adjusting the amine percentage in the PBZs structure.^45^ Wan et al. revealed that NPMCs with high O and N contents derived from BZ with CN groups showed a maximum capacitance of 362.4 F g^–1^, along with an impressive retention rate of 94.7%.^47^

Based on the information above, we constructed and prepared poly(Cr-TPA-4BZ-Py-POP)-800 N-doped microporous carbon through the carbonization and KOH activation for the poly(Cr-TPA-4BZ-Py-POP) sample at 800 °C. First, to produce the Cr-TPA-4BZ-Br_4_ monomer, the classic three-step procedure [involving CH=N formation, reduction by NaBH_4_, and Mannich condensation] was employed. Subsequently, the Cr-TPA-4BZ-Br_4_ and Py-T monomers underwent Sonogashira coupling to form Cr-TPA-4BZ-Py-POP. The successful synthesis of Cr-TPA-4BZ-Br_4_ and Cr-TPA-4BZ-Py-POP was confirmed using analytical methods such as differential scanning calorimetry (DSC), thermogravimetric analysis (TGA), Fourier transform infrared (FTIR), and solid-state ^13^C NMR. poly(Cr-TPA-4BZ-Py-POP)-800 demonstrated a significant specific capacitance value of 397.2 F g^–1^ and CO_2_ of 4.4 mmol/g in practical applications. The exceptional performance of poly(Cr-TPA-4BZ-Py-POP)-800 N-doped microporous carbon holds promise for substantial contributions to addressing urgent challenges in both energy storage, gas capture and environmental applications.

## Experimental Section

### Materials

The specified materials were sourced from Sigma-Aldrich, including salicylaldehyde (SA-CHO), 4-bromosalicylaldehyde (SA-CHO-Br, 97%), hydrazine monohydrate (NH_2_NH_2_·H_2_O, ≥97%), sodium borohydride (NaBH_4_, ≥98.0%), sodium hydroxide (NaOH, ≥98.0%), catechol (98%), dimethyl sulfoxide (DMSO, ≥99.9%), absolute ethanol (EtOH, ≥99.5%), methanol (MeOH, ≥98%), *N*,*N*-dimethylformamide (DMF), toluene (99.8%), acetic acid (AcOH, 99.8%), triphenylphosphine (PPh_3_), tetrakis(triphenylphosphine)palladium(0) (Pd(PPh_3_)_4_, 99.99%), copper powder (Cu, 99.999%), anhydrous MgSO_4_ (≥97%), copper(I) iodide (CuI, 99.999%), bis(2-chloroethyl)ether (≥99.0%), and CH_2_Cl_2_. Acros supplied additional materials, including paraformaldehyde (CH_2_O)*_n_*, 1,4-dioxane (DO, 99.8%), hydrochloric acid (HCl, 37%), nitric acid (HNO_3_, 65%), palladium on activated carbon (Pd/C, 10 wt %), and acetone. The synthesis of Py-T and Cr-TPA-4NH_2_ adhered to established protocols [Schemes S1 and S2].^48−52^

### Synthesis of Cr-TPA-4SF

A solution containing Cr-TPA-4NH_2_ (3 g, 4 mmol) and SA-CHO (1.94 g, 16 mmol) in 100% ethanol (150 mL) underwent reflux for 24 h at 85 °C. After cooling, the orange compound was filtered, washed with ethanol, and kept at 40 °C for 1 day, yielding 85%. FTIR (KBr): 3419 (OH stretching) cm^–1^. ^1^H NMR (500 MHz, DMSO-*d*_6_): 13.11 (OH), 9.03 (NH).

### Synthesis of Cr-TPA-4RED

After Cr-TPA-4SF (2.5 g, 2.13 mmol) was dissolved in 70 mL of EtOH at room temperature, 0.81 g (21.11 mmol) of NaBH_4_ was added to the mixture, which was then shaken in a 100 mL flask for a day at 50 °C. Subsequently, the mixture was transferred to 300 mL of ice-cold water. Following filtration, the gray solid underwent three water washes and was then dried, resulting in a yield of 95%. FTIR (KBr): 3291 (NH stretching). ^1^H NMR (500 MHz, DMSO-*d*_6_): 5.76 (NH), 4.1 (NH–CH_2_).

### Synthesis of Cr-TPA-4BZ

Subjected to a one-day treatment at 110 °C under a nitrogen atmosphere, Cr-TPA-4RED (3 g, 2.54 mmol) and (CH_2_O)*_n_* (0.10 g, 12.72 mmol) were heated in a 250 mL flask and dissolved in a solution of DO (80 mL) and EtOH (20 mL). Upon cooling, the solvents evaporated, and the resulting mixture was cleansed three times with MeOH, yielding a yellow solid (80%). FTIR (KBr): 2930, 2867, 1270 (asymmetric C–O–C stretching), 942 (oxazine ring) cm^–1^. ^1^H NMR (500 MHz, DMSO-*d*_6_): 7.08–6.73 (aromatic protons), 5.38 (OCH_2_N), and 4.57 (ArCH_2_N) ppm.

### Synthesis of Cr-TPA-4SF-Br_4_

A solution containing Cr-TPA-4NH_2_ (1.00 g, 1.32 mmol) and SA-CHO-Br (1.06 g, 5.29 mmol) in 100% ethanol (110 mL) underwent reflux for 24 h at 85 °C. After it was cooled, the orange solid was filtered, washed [by ethanol], and kept at 40 °C for a day, yielding 80%. The FTIR (KBr) spectrum revealed the following peaks: 3373 (OH stretching), 3063 (C=C–H), 1617 (C–N), 1128 (C–O–C), and 596 (C–Br) cm^–1^. The ^1^H NMR (500 MHz, DMSO-*d*_6_) spectrum exhibited signals at δ = 5.07 (OH), 8.97 (N–CH), and 7.5–6.5 (aromatic protons) ppm.

### Synthesis of Cr-TPA-4RED-Br_4_

After Cr-TPA-4SF-Br_4_ (1.00 g, 0.85 mmol) was dissolved in 40 mL of EtOH at room temperature, 0.13 g (3.40 mmol) of NaBH_4_ was added to the mixture, which was then shaken in a 100 mL flask for a day at 50 °C. Subsequently, the mixture was transferred to 300 mL of ice-cold water. Following filtration, the gray solid underwent three water washes and was then dried, resulting in a yield of 90%. FTIR (KBr): 3287 (NH stretching) and 3404 (OH stretching) cm^–1^. ^1^H NMR (500 MHz, DMSO-*d*_6_): 5.38 (OH), 4.54 (NH), and 7.14–6.28 (aromatic protons) ppm.

### Synthesis of Cr-TPA-4BZ-Br_4_

Subjected to a one-day treatment at 110 °C under a nitrogen atmosphere, Cr-TPA-4RED-Br_4_ (1 g, 0.66 mmol) and (CH_2_O)*_n_* (0.10 g, 3.34 mmol) were heated in a 250 mL flask and dissolved in a solution of DO (100 mL)/EtOH (40 mL). Upon cooling, the solvents evaporated, and the resulting mixture was cleaned three times with MeOH, yielding a yellow solid (90%). FTIR (KBr): 2927, 2881, 1263 (asymmetric C–O–C stretching), and 943 (oxazine ring) cm^–1^. ^1^H NMR (500 MHz, DMSO-*d*_6_): 7.03–6.50 (aromatic protons), 5.40 (OCH_2_N), and 4.56 (ArCH_2_N) ppm.

### Synthesis of Cr-TPA-4BZ-Py-POP

In a Pyrex tube, DMF (10 mL) and Et_3_N (10 mL) were introduced to a mixture containing Cr-TPA-4BZ-Br_4_ (0.2 g, 0.130 mmol), Py-T (33 mg, 0.130 mmol), CuI (3 mg), PPh_3_ (5 mg), and Pd(PPh_3_)_4_ (1.5 mg, 0.013 mmol). Following three cycles of freeze, pump, and thaw, the resulting liquid was homogenized and then heated to 110 °C for 3 days. The mixture underwent filtration and subsequent washing with MeOH, and acetone, as part of the Sonogashira coupling process to form Cr-TPA-4BZ-Py-POP as a red powder with a yield of 95%.

### Thermal Polymerization of the Cr-TPA-4BZ, Cr-TPA-4BZ-Br_4_, and Cr-TPA-4BZ-Py-POP

For the synthesis of poly(Cr-TPA-4BZ) and poly(Cr-TPA-4BZ-Br_4_), both Cr-TPA-4BZ and Cr-TPA-4BZ-Br_4_ underwent heating cycles at 110, 150, 280, and 210 °C, each lasting 3 h. In the case of poly(Cr-TPA-4BZ-Py-POP), the corresponding poly(Cr-TPA-4BZ-Py-POP) was subjected to heating at 110, 150, 180, and 210 °C, with each temperature maintained for 3 h.

### Formation of Poly(Cr-TPA-4BZ-Py-POP)-700 and Poly(Cr-TPA-4BZ-Py-POP)-800 N-Doped Microporous Carbon

After the poly(Cr-TPA-4BZ-Py-POP) sample underwent full thermal curing at 210 °C, it was placed in a tube furnace and heated at 10 °C min^–1^ until it reached 600 °C. It was then calcined at this high temperature for 6 h. After the calcination process, the sample was allowed to cool naturally before being removed from the furnace. Subsequently, the calcined sample was mixed with an aqueous KOH solution (KOH/H_2_O, 1:1, w/w) and stirred for 24 h at room temperature. Following the removal of water, the material was activated in a tube furnace under a N_2_ flow for 8 h at 700 and 800 °C. The materials were repeatedly washed with deionized water and the pH was checked using litmus paper until a neutral pH of 7 was achieved. Afterward, the materials were dried in an oven at 120 °C.

## Results and Discussion

### Synthesis and Characterization of Cr-TPA-4BZ and Cr-TPA-4BZ-Br_4_ Monomers

**Figure 1:**
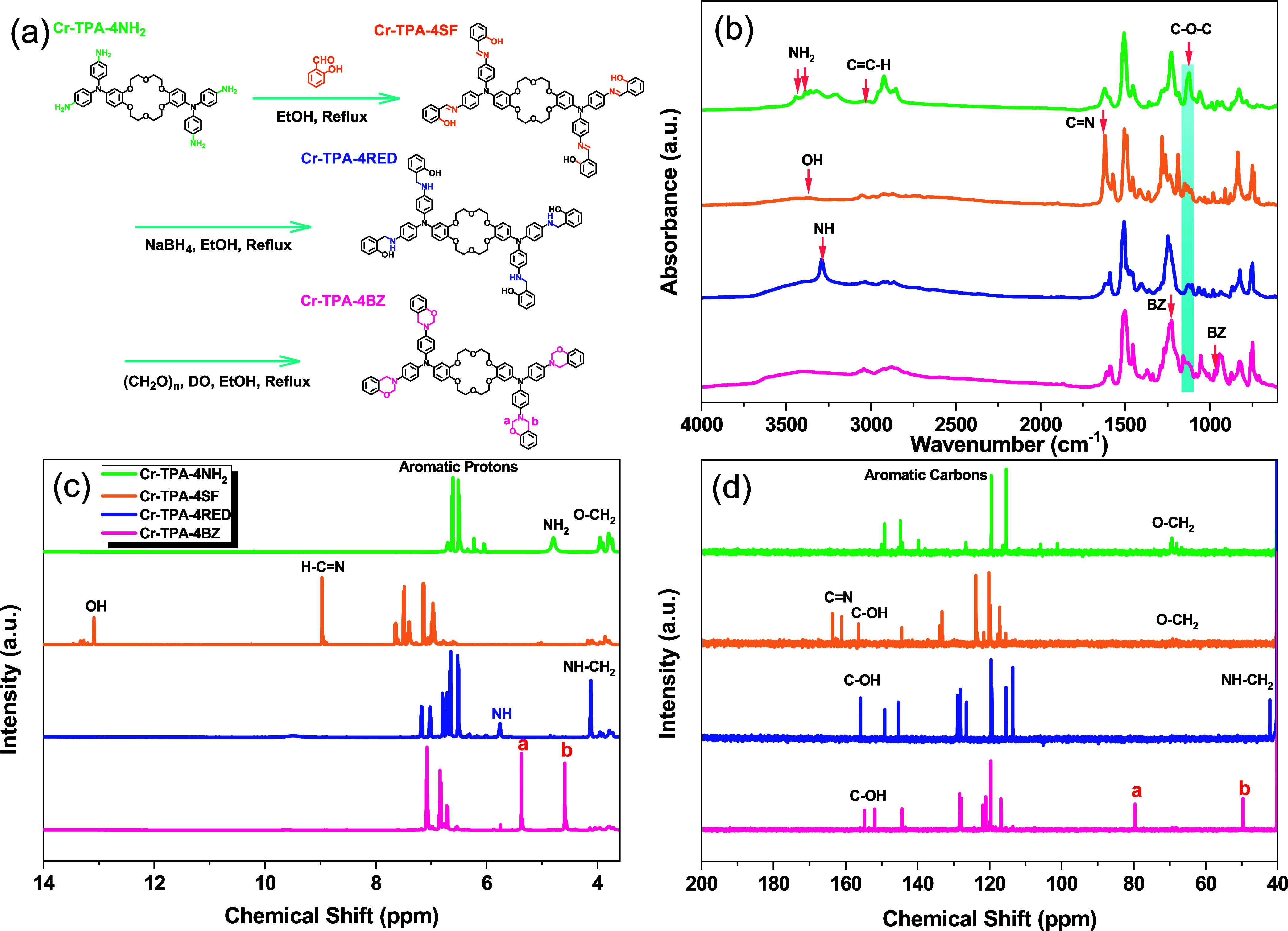
(a) Synthesis process of Cr-TPA-4SF, Cr-TPA-4RED and Cr-TPA-4BZ from Cr-TPA-4NH_2_. (b) FTIR, (c) ^1^H NMR, and (d) ^13^C NMR spectra of Cr-TPA-4NH_2_, Cr-TPA-4SF, Cr-TPA-4RED and Cr-TPA-4BZ.

In our study, achieving a high degree of cross-linking in POPs necessitates the synthesis of a benzoxazine (BZ) monomer with exceptional purity. Given that a one-step synthesis method tends to be fast but compromises purity, we opted for a meticulous three-step synthesis approach. This method involves the sequential processes of Schiff base reaction, reduction, and Mannich condensation reaction to obtain our desired Cr-TPA-4BZ monomer, serving as a model compound. The synthesis process of the Cr-TPA-4BZ monomer is illustrated in Figure 1(a), while Figure 1(b) depicts the FTIR pattern of the Cr-TPA-4BZ monomer. Notably, in the FTIR spectrum of Cr-TPA-4NH_2_, characteristic signals at 3387, 3358, and 1124 cm^–1^ correspond to NH_2_ and C–O–C (crown ether), respectively. Following the Schiff base reaction signals, the values at 3373 and 1619 cm^–1^ signify phenolic–OH and C=N groups, respectively. The FTIR spectrum of Cr-TPA-4RED exhibits bands at 3381 cm^–1^ [OH] and 3290 cm^–1^ [NH group], while the C=N peak diminishes due to its transformation to the −NH unit. After Mannich condensation of Cr-TPA-4RED to form Cr-TPA-4BZ, the bands at 942 and 1270 cm^–1^ appear, indicating the presence of an oxazine ring and C–O–C stretching, respectively. Figure 1(c,d) presents the ^1^H and ^13^C NMR patterns of the Cr-TPA-4NH_2_, Cr-TPA-4RED, and Cr-TPA-4BZ. The ^1^H NMR spectrum of Cr-TPA-4NH_2_ displays signals for the aromatic ring, NH_2_, and O–CH_2_ (crown ether) at 6.78–5.98, 4.797, and 4.09–3.67 ppm, respectively. Post Schiff base reaction to afford Cr-TPA-4SF, OH, H–C=N, aromatic rings, and O–CH_2_ signals are observed at 13.09, 8.97, 7.65–6.57, and 4.2–3.7 ppm. Reduction of Cr-TPA-4SF with NaBH_4_ results in the appearance of signals at 5.76 and 4.11 representing NH and NH–CH_2_ groups, with the disappearance of the C=N signal, indicating the successful synthesis of Cr-TPA-4RED. In the Mannich condensation reaction of Cr-TPA-4RED to produce Cr-TPA-4BZ, the ^1^H NMR profile exhibited characteristic signals at 5.37 (OCH_2_N) and 4.59 (ArCH_2_N) ppm, with a near 1:1 ratio. The signal of NH–CH_2_ disappears, confirming the success of the synthesis of Cr-TPA-4BZ. The ^13^C NMR spectrum of Cr-TPA-4NH_2_ reveals signals for aromatic carbons and O–CH_2_ (crown ether) at 149.96–101.17 and 69.44 ppm. Following the Schiff base reaction to form Cr-TPA-4SF, signals for C=N, C–OH, aromatic carbons, and O–CH_2_ appear at 163.65, 156.42, 144.21–115.49, and 69.7–67.5 ppm, respectively. The reduction reaction of Cr-TPA-4SF to Cr-TPA-4RED produces signals at 155.83 and 42.16 ppm for C–OH and NH–CH_2_, respectively, with the disappearance of C=NH. Subsequently, the ring closing in Cr-TPA-4BZ reveals signals for the oxazine ring at 79.61 ppm [O–CH_2_–N] and 49.63 [Ar–CH_2_–N] ppm, respectively, and the NH–CH_2_ signal disappears, confirming the creation of the Cr-TPA-4BZ material.

**Figure 2:**
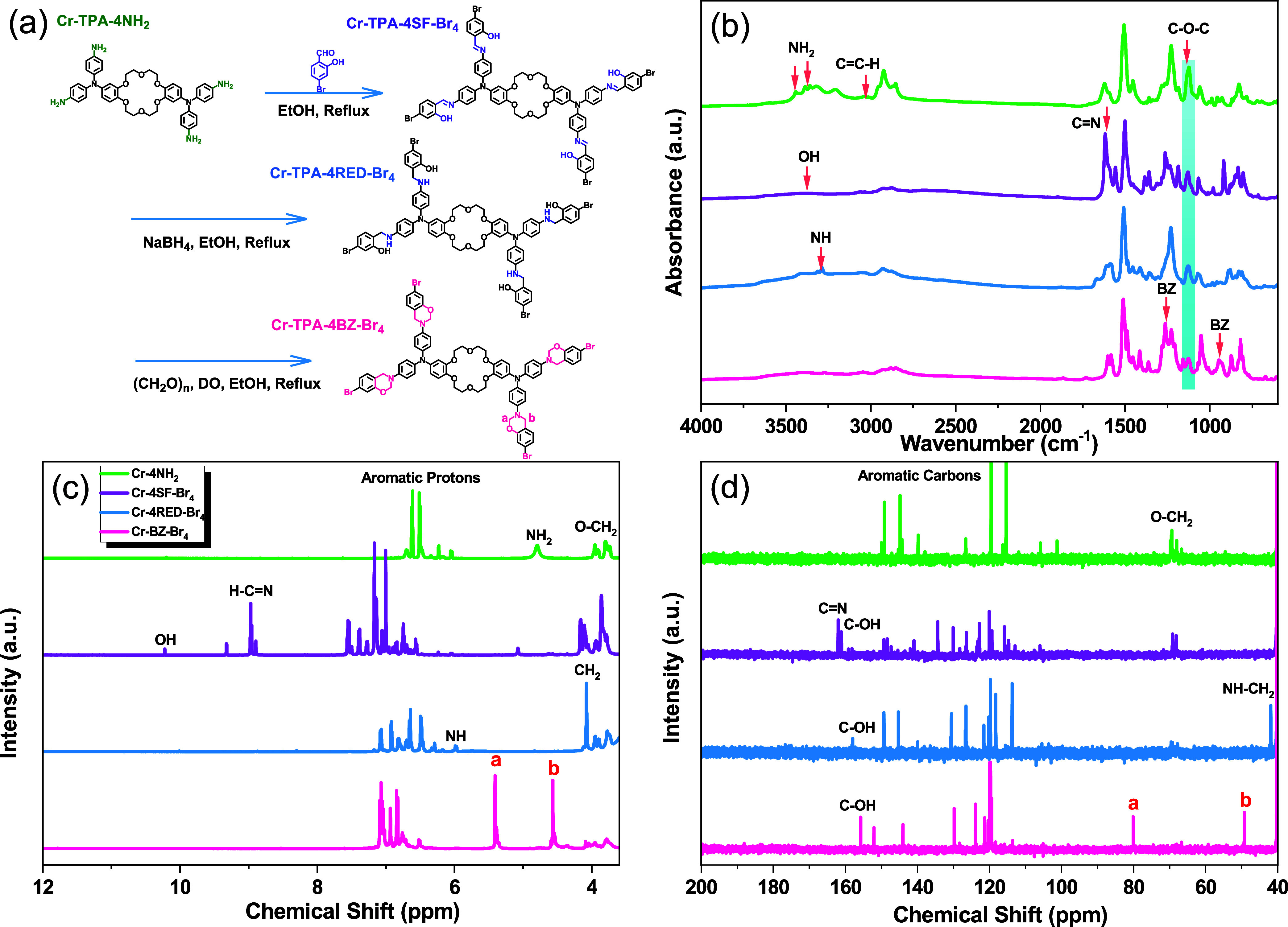
(a) Synthesis process of Cr-TPA-4SF-Br_4_, Cr-TPA-4RED-Br_4_, and Cr-TPA-4BZ-Br_4_ from Cr-TPA-4NH_2_. (b) FTIR, (c) ^1^H NMR, and (d) ^13^C NMR spectra of Cr-TPA-4NH_2_, Cr-TPA-4SF-Br_4_, Cr-TPA-4RED-Br_4_, and Cr-TPA-4BZ-Br_4_.

In the synthesis process depicted in Figure 2(a), Cr-TPA-4BZ-Br_4_ was produced using a method analogous to that of Cr-TPA-4BZ, with Cr-TPA-4NH_2_ serving as the precursor. However, a modification was made by substituting 4-bromosalicylaldehyde (SA-CHO-Br) as the intermediate reactant in the Schiff base reaction to form Cr-TPA-4SF-Br_4_. Subsequently, a reduction reaction of Cr-TPA-4SF-Br_4_ with NaBH_4_ transformed it into Cr-TPA-4RED-Br_4_, followed by a Mannich contraction reaction for ring closure to yield the final Cr-TPA-4BZ-Br_4_ monomer. The FTIR spectrum presented in Figure 2(b) reveals distinctive features of Cr-TPA-4SF-Br_4_, Cr-TPA-4RED-Br_4_, and Cr-TPA-4BZ-Br_4_. The signals at 3370, 1618, and 596 cm^–1^ after CH=N formation denote the presence of phenolic–OH, C=N, and C–Br groups in the Cr-TPA-4SF-Br_4_. Upon examination of the FTIR spectrum of Cr-TPA-4RED-Br_4_, the peaks at 3404 and 3287 cm^–1^ correspond to OH and NH, while the C=N peak diminishes due to its conversion to NH unit. Following the Mannich condensation reaction of Cr-TPA-4RED-Br_4_, Cr-TPA-4BZ-Br_4_ exhibits absorption peaks at 3405, 1263, and 943 cm^–1^, indicative of the presence of OH, oxazine ring, and C–O–C stretching, respectively. Figure 2(c,b) showcases the ^1^H and ^13^C NMR spectra of each compound at room temperature. After the Schiff base reaction, the Cr-TPA-4SF-Br_4_ spectrum reveals peaks at 10.22, 9.32, 9.04–6.02, and 4.22–3.66 ppm corresponding to OH, H–C=N, aromatic rings, and O–CH_2_ in the crown ether ring. Reduction of Cr-TPA-4SF-Br_4_ to Cr-TPA-4RED-Br_4_ results in characteristic peaks at 157.98 and 41.93 ppm for C–OH and NH–CH_2_, respectively. Mannich condensation reaction on Cr-TPA-4RED-Br_4_ yields Cr-TPA-4BZ-Br_4_, where distinctive signals at 4.41 (ArCH_2_N) and 4.57 (OCH_2_N) ppm confirm the successful synthesis of Cr-TPA-4BZ-Br_4_, with the NH–CH_2_ unit disappearing. The ^13^C NMR profiles [Figure 2(d)] showed signals at 162, 161.08, 159.15–105.01, and 69 ppm, respectively, due to C–OH, C=N, aromatic carbons, and O–CH_2_ in the Cr-TPA-4SF-Br_4_. They were 157.9 and 41.9 ppm, representing C–OH and NH–CH_2_ groups in the Cr-TPA-4RED-Br_4_, and they were 80 (OCH_2_N) and 49.22 (ArCH_2_N) ppm in the Cr-TPA-4BZ-Br_4_ structure.

### Thermal Polymerization Behavior of Cr-TPA-4BZ and Cr-TPA-4BZ-Br_4_

**Figure 3:**
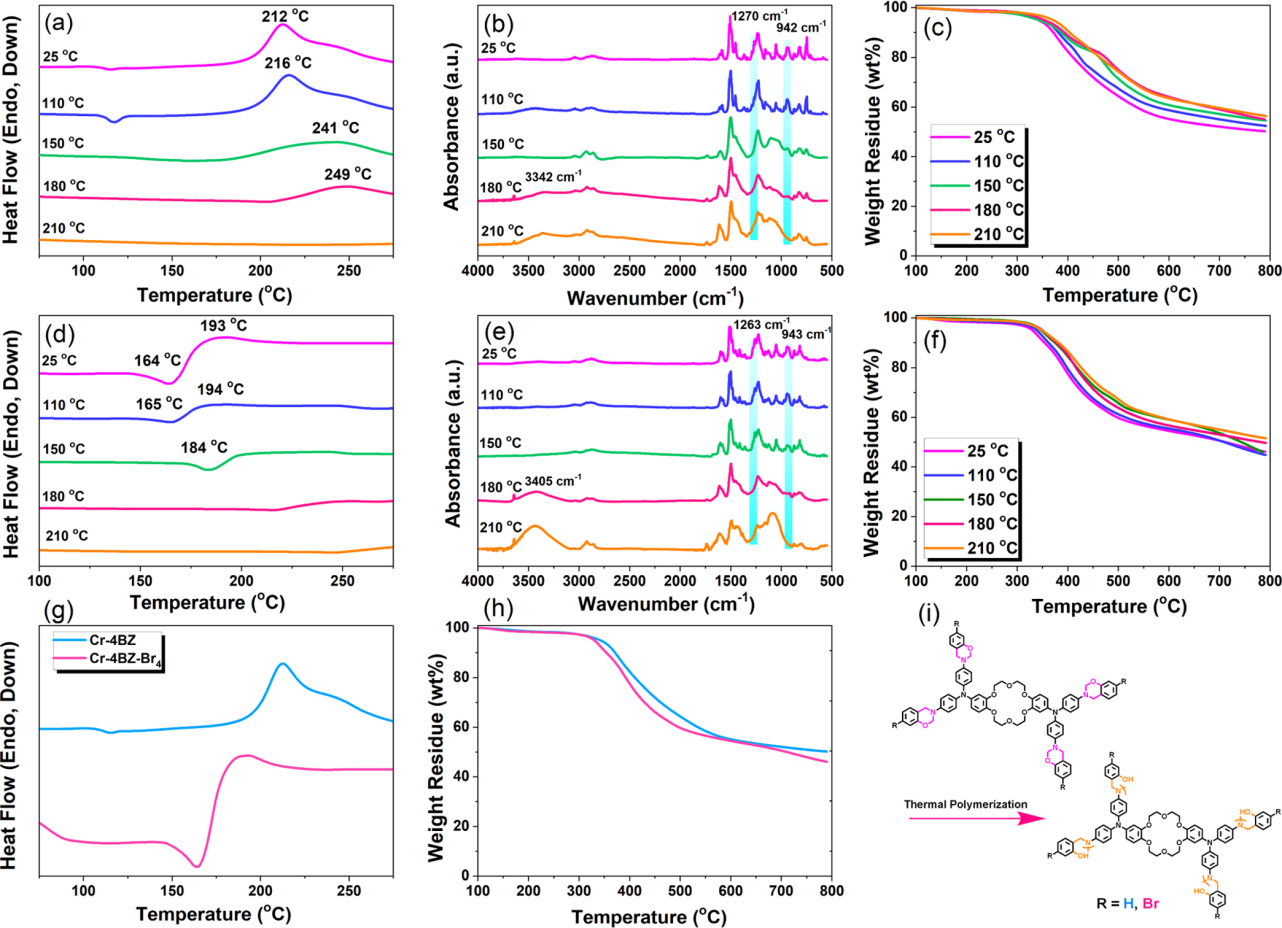
(a) DSC, (b) FTIR, and (c) TGA for Cr-TPA-4BZ monomer; (d) DSC, (e) FTIR, and (f) TGA for Cr-TPA-4BZ-Br_4_ monomer undergo various temperature thermal polymerization; (g, h) DSC and TGA comparison between Cr-TPA-4BZ and Cr-TPA-4BZ-Br_4_ monomers; and (i) schematic diagram of solid-state chemical transformation behavior of Cr-TPA-4BZ and Cr-TPA-4BZ-Br_4_ monomers.

The thermal curing of Cr-TPA-4BZ and Cr-TPA-4BZ-Br_4_ monomers was investigated by using DSC, FTIR, and TGA at various heating stages. In Figure 3(a), a prominent endothermic thermal polymerization peak at 212.6 °C signifies the synthesis of a high-purity Cr-TPA-4BZ monomer. With increasing temperature during heat treatment, both endothermic and exothermic peaks shift rightward and decrease in intensity. After 3 h at 210 °C, both peaks nearly vanish, indicating complete thermal ring-opening behavior. Figure 3(b) presents the FTIR spectrum aligned with the DSC findings. Special attention is given to the C–O–C group and oxazine ring signals at 1270 and 942 cm^–1^, which gradually diminish with temperature elevation until their disappearance at 210 °C. Concurrently, the appearance of the OH group at 3342 cm^–1^ confirms the thermal curing of the Cr-TPA-4BZ. TGA, illustrated in Figure 3(c), shows that uncured Cr-TPA-4BZ exhibits T_d10_ and char yield values of 372 °C and 50.3%, respectively. After thermal treatment of Cr-TPA-4BZ for 3 h at 210 °C, Cr-TPA-4BZ displays increased thermal stability with T_d10_ and char yield values of 399 °C and 56.23%, affirming the high char yield attributed to cross-linking via hydrogen bonds between phenolic OH groups and nitrogen atoms in Mannich bridges. Figure 3(d) reveals two thermal phenomena for Cr-TPA-4BZ-Br_4_: a melting exothermic peak at 164.1 °C and an endothermic thermal polymerization peak at 193.3 °C. Similar to Cr-TPA-4BZ, both peaks shift rightward and diminish with increasing temperature during heat treatment, disappearing after 3 h at 210 °C, confirming the complete thermal ring-opening behavior of Cr-TPA-4BZ-Br_4_. Figure 3(e) depicts the FTIR spectrum of Cr-TPA-4BZ-Br_4_ to monitor the ROP during thermal treatments. As observed in Figure 3(c), the C–O–C group and oxazine ring signals at 1263 and 943 cm^–1^ gradually diminish with rising temperature until disappearance at 210 °C. In Figure 3(f), TGA shows that the uncured Cr-TPA-4BZ-Br_4_ has T_d10_ and char yield values of 354 °C and 46.14%. After thermal curing at 210 °C for 2 h, Cr-TPA-4BZ-Br_4_ exhibits enhanced thermal stability with T_d10_ and char yield values of 382 °C and 52%. Figure 3(g) compares the DSC curves of Cr-TPA-4BZ and Cr-TPA-4BZ-Br_4_, revealing that the replacement of H with Br shifts the endothermic thermal polymerization peak from 212.6 to 193.3 °C, shortening the overall melting and curing process. This is attributed to the electron-absorbing effect of the Br atom, inducing a pulling effect that facilitates ring opening. The same trend is observed in TGA [Figure 3(h)], where Br substitution results in decreased T_d10_ and char yield of Cr-TPA-4BZ-Br_4_ due to the electron-pulling effect of the Br atom. Figure 3(i) illustrates the final molecular chemical structures of poly(Cr-TPA-4BZ) and poly(Cr-TPA-4BZ-Br_4_) after ROP at 210 °C.

### Synthesis and Thermal Polymerization Behavior of Cr-TPA-4BZ-Py-POP

**Figure 4:**
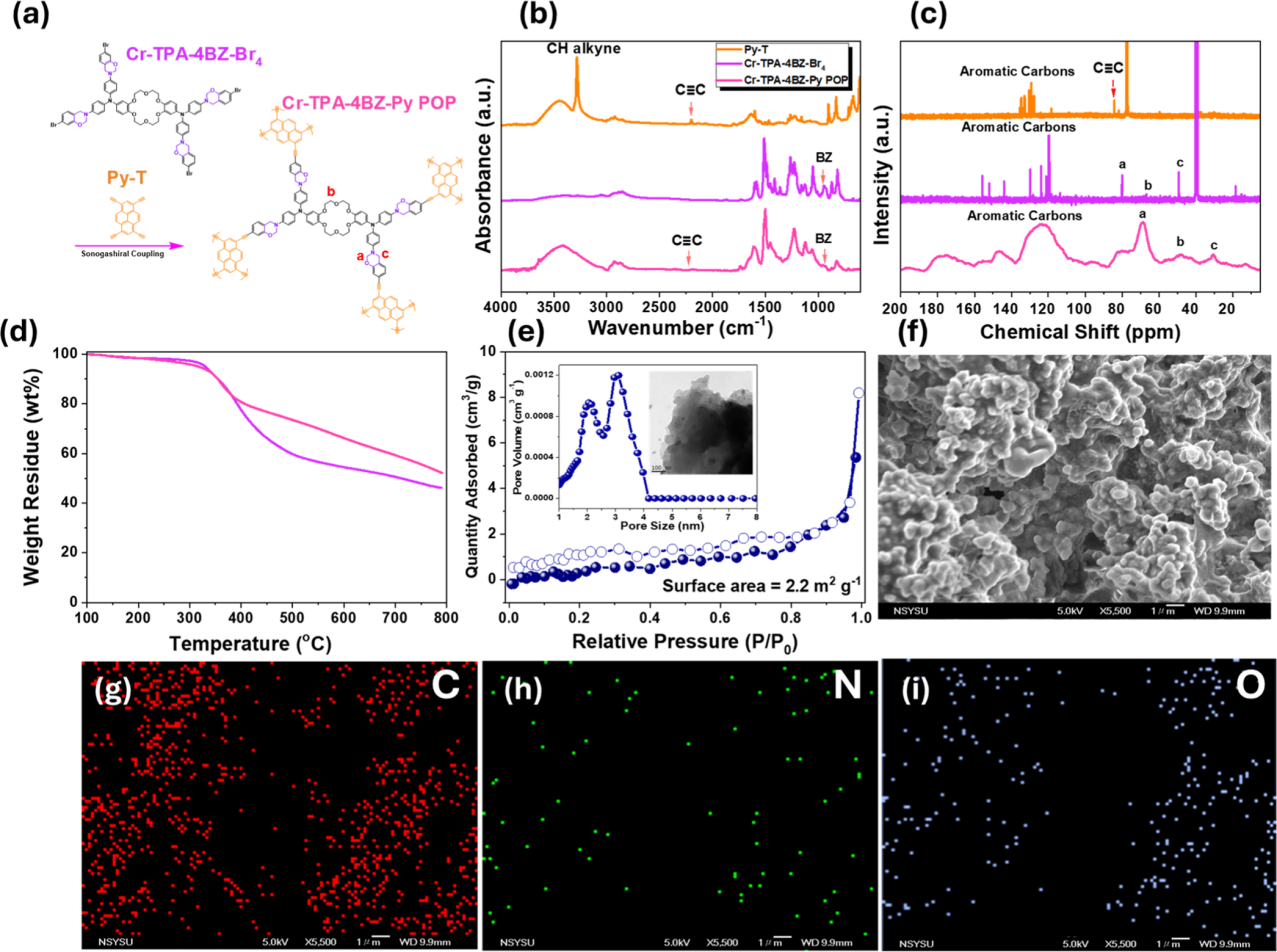
(a) Schematic diagram of Cr-TPA-4BZ-Py-POP, (b) FTIR and (c) ^13^C NMR and SSNMR data of Py-T, Cr-TPA-4BZ-Br_4_, and Cr-TPA-4BZ-Py-POP, (d) TGA and (e) N_2_ adsorption/desorption isotherms curve, pore size, and TEM image, (f) SEM images and (g–i) SEM-EDS mapping of Cr-TPA-4BZ-Py-POP.

In the synthesis of Cr-TPA-4BZ-Py-POP, the Cr-TPA-4BZ-Br_4_ monomer and Py-T were employed as the basic building blocks, with Pd(PPh_3_)_4_ serving as the catalyst. A cosolvent comprising a 1:1 ratio of DMF/Et_3_N was utilized, and the reaction was conducted at 110 °C for 3 days, leading to the successful development of Cr-TPA-4BZ-Py-POP as a red solid through the Sonogashira coupling reaction [Figure 4(a)]. The FTIR analysis [Figure 4(b)] revealed distinct signals in Py-T at 3281 and 2199 cm^–1^, signifying C≡C–H and C≡C units. Similarly, Cr-TPA-4BZ-Br_4_ exhibited signals at 943 and 1263 cm^–1^ due to the oxazine and C–O–C units, respectively. Following the Sonogashira coupling reaction, the disappearance of the C≡C–H signal and the appearance of signals at 2185, 1228, and 959 cm^–1^ indicated the successful synthesis of Cr-TPA-4BZ-Py-POP. The ^13^C NMR spectrum displayed distinct peaks for Py-T and Cr-TPA-4BZ-Br_4_, confirming the presence of aromatic carbons, C≡C, and oxazine groups [Figure 4(c)]. Solid-state NMR further validated the synthesis of Cr-TPA-4BZ-Py-POP by showcasing characteristic signals at 82.36, 80.06, 68.72, and 48.72 ppm for C≡C, carbons atoms in the Cr unit, and the oxazine ring, respectively. Thermogravimetric analysis [Figure 4(d)] demonstrated a higher char yield for Cr-TPA-4BZ-Py-POP (52.2%) compared to the Cr-TPA-4BZ-Br_4_ monomer (46.1%), indicating improved thermal stability after the Sonogashira coupling reaction. BET adsorption and desorption curves [Figure 4(e)] under a nitrogen flow at 77 K revealed an *S*_BET_ of 2.27 m^2^ g^–1^ and a total pore volume (*V*_t_) of 0.012 cm^3^ g^–1^ for Cr-TPA-4BZ-Py-POP. The isotherm curve exhibited a type I classification, suggesting microporous structures. The lower *S*_BET_ of Cr-TPA-4BZ-Py-POP may be attributed to the inherent flexibility of the crown ether (Cr) central unit, and the reduced surface area of Cr-TPA-4BZ-Py-POP may be attributed to the pronounced π–π interaction between electron-rich Py units, facilitated by a flexible Cr linkage. Zhou and Chi et al. have reported a low *S*_BET_ for Cr-based POPs aligning with our collected data.^53,54^ Nonlocal density functional theory (NLDFT) was employed to ascertain the distribution of pore sizes, revealing micro-mesopores in the range of about 0.39–4.17 nm. High-resolution transmission electron microscopy (HR-TEM) and scanning electron microscopy (SEM) images of Cr-TPA-4BZ-Py-POP [Figure 4(e,f)] illustrated no long-range order and irregular and disordered spherical morphology. Energy-dispersive X-ray spectroscopy (EDS) images [Figure 4(g–i)] display the distribution and proportion of carbon, nitrogen, and oxygen in the Cr-TPA-4BZ-Py-POP sample. In conclusion, Cr-TPA-4BZ-Py-POP was successfully synthesized, and its structural and thermal properties, as well as porosity, were thoroughly characterized through various analytical techniques.

**Figure 5:**
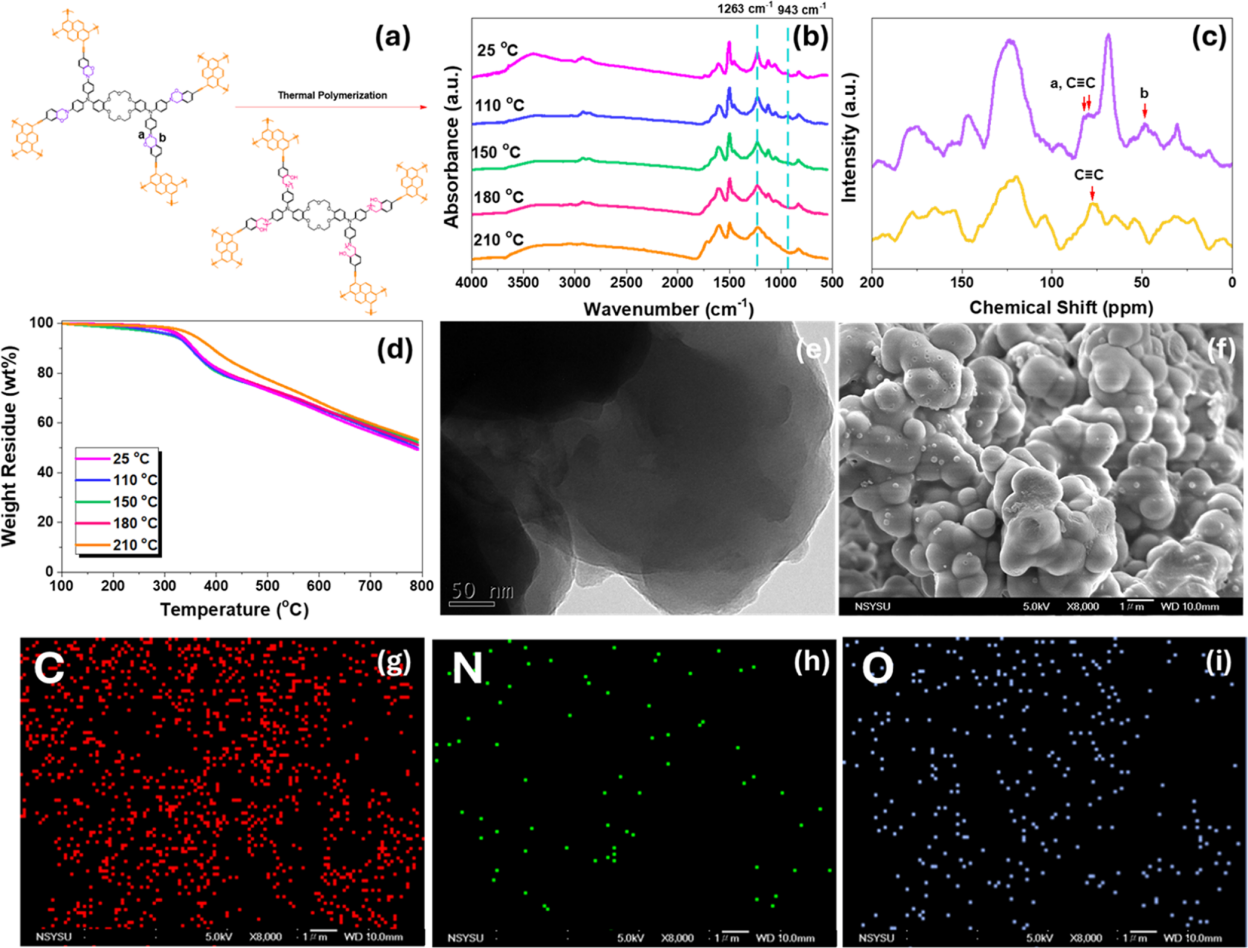
(a) Schematic diagram of solid-state chemical transformation behavior of Cr-TPA-4BZ-Py-POP at 210 °C to form poly(Cr-TPA-4BZ-Py-POP); (b) FTIR, (c) SSNMR, (d) TGA, (e) TEM image, (f) SEM images, and (g–i) SEM-EDS mapping of poly(Cr-TPA-4BZ-Py-POP).

**Figure 6:**
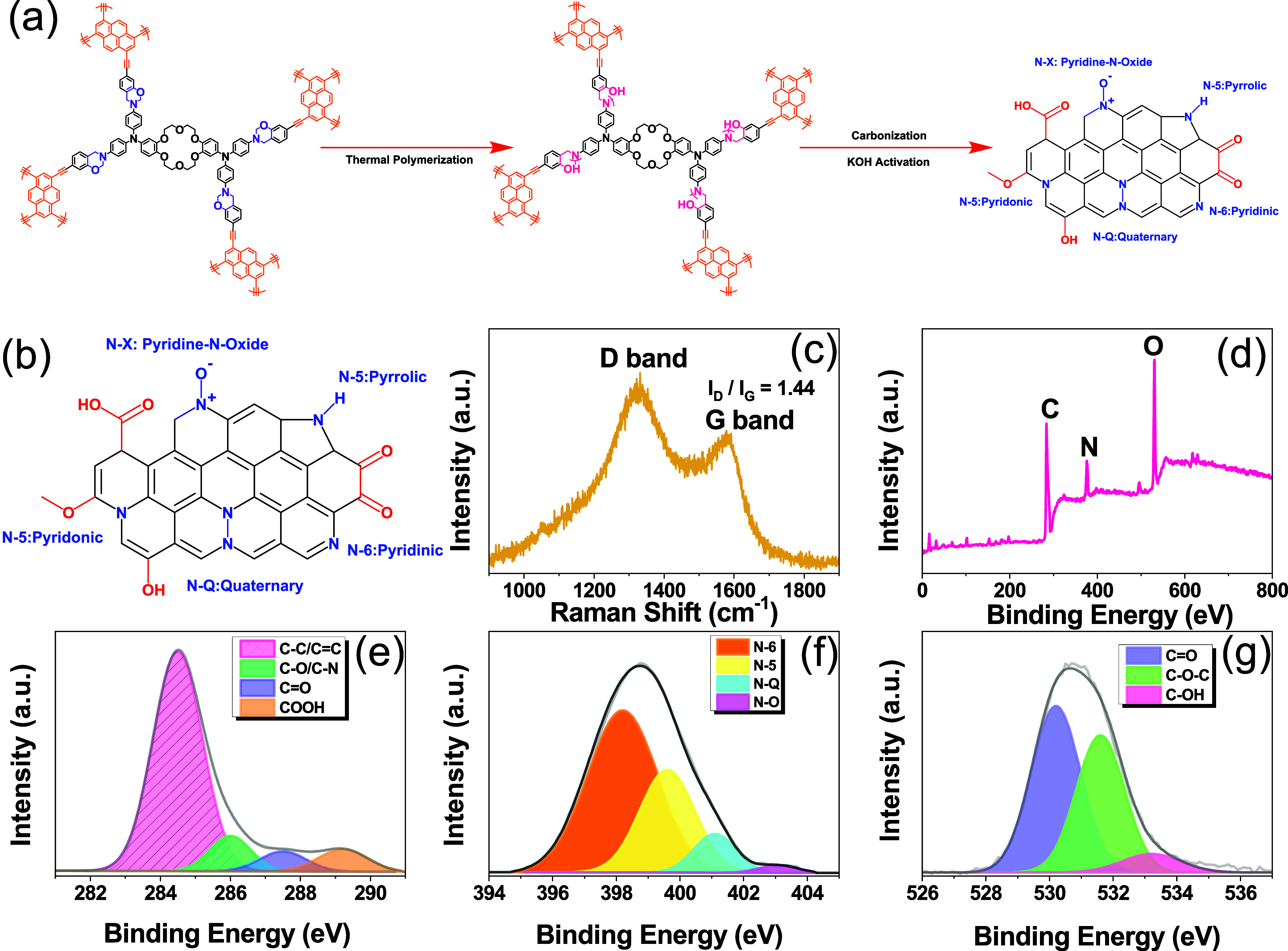
(a) Schematic diagram for the formation poly(Cr-TPA-4BZ-Py-POP)-800 through thermal polymerization, carbonization, and KOH activation of Cr-TPA-4BZ-Py-POP and poly(Cr-TPA-4BZ-Py-POP), (b) chemical structure of poly(Cr-TPA-4BZ-Py-POP)-800, (c) Raman spectra and XPS spectra, (d) Survey (e) C 1s, (f) N 1s, and (g) O 1s orbitals for poly(Cr-TPA-4BZ-Py-POP)-800.

Figure 5(a) shows a schematic illustration depicting the solid-state chemical transformation [SSCT] behavior of Cr-TPA-4BZ-Py-POP through ROP at 210 °C, resulting in the formation of poly(Cr-TPA-4BZ-Py-POP). Subsequently, Figure 5(b) presents the FTIR spectra of Cr-TPA-4BZ-Py-POP under ROP at different temperatures for 3 h. Notably, the BZ ring in the Cr-TPA-4BZ-Py-POP signals gradually diminishes and disappears at positions 1228 and 959 cm^–1^, affirming the successful execution of the ROP with a significant impact. A significant peak spanning the range of 3684–1828 cm^–1^ suggests the formation of hydrogen bonding forces due to OH groups after ring-opening polymerization of BZ units in the Cr-TPA-4BZ-Py-POP. Solid-state NMR analysis [Figure 5(c)] further supports the ROP-induced changes, with characteristic peaks of OCH_2_N and ArCH_2_N in the BZ ring at 80.06 and 48.72 ppm gradually decreased after thermal treatments at 210 °C for 3 h, providing evidence of the complete ROP of the Cr-TPA-4BZ-Py-POP to form the poly(Cr-TPA-4BZ-Py-POP) framework. The impact of ROP on thermal properties is elucidated through TGA in Figure 5(d). The uncured Cr-TPA-4BZ-Py-POP exhibits a T_d10_ of 354 °C and a char yield of 52.16 wt %, whereas poly-Cr-4BZ-Py-POP after ROP displays a substantial increase to 393 °C and 53 wt %, respectively. These findings highlight the achievement of a highly cross-linked state through solid-state chemical transformation, underscoring the positive influence of the chemical structure on the enhanced thermal properties. Figure 5(e) showcases HR-TEM images of poly(Cr-TPA-4BZ-Py-POP), revealing the bright and dark areas that signify irregularity and disorder. Correspondingly, SEM images [Figure 5(f)] of poly(Cr-TPA-4BZ-Py-POP), emphasize the irregular spherical shape and disordered arrangement of the poly(Cr-TPA-4BZ-Py-POP) framework. The EDS images [Figure 5(g–i)] of poly(Cr-TPA-4BZ-Py-POP) further characterize the distribution and proportion of carbon, nitrogen, and oxygen elements. In summary, the presented figures collectively demonstrate the successful SSCT of Cr-TPA-4BZ-Py-POP through thermal ROP, with comprehensive analyses validating the structural and thermal changes, as well as morphological alterations, in the resulting poly(Cr-TPA-4BZ-Py-POP). Figure 6(a) outlines the carbonization and KOH activation process for creating poly(Cr-TPA-4BZ-Py-POP)-800 N-doped microporous carbon. The procedure is initiated with an ROP of Cr-TPA-4BZ-Py-POP at 210 °C to establish a robust cross-linking network [poly(Cr-TPA-4BZ-Py-POP)] and carbonized poly(Cr-TPA-4BZ-Py-POP) at 600 °C for 2 h. Then, the poly(Cr-TPA-4BZ-Py-POP)-600 powder is immersed in a 3 M KOH aqueous solution, mixed for 24 h, and subjected to an 800 °C tube furnace for 8 h. The resulting product is poly(Cr-TPA-4BZ-Py-POP)-800 [Figure 6(b)]. Figure S1 presents the XRD analysis of poly(Cr-TPA-4BZ-Py-POP)-800, revealing distinct diffraction peaks at 2θ = 25.8 and 43° corresponding to (200) and (100) graphitic planes of carbon, respectively.^55^Figure 6(c) displays the Raman spectrum (1000–1900 cm^–1^), featuring D and G bands at approximately 1350 and 1580 cm^–1^. The *I*_D_/*I*_G_ value of poly(Cr-TPA-4BZ-Py-POP)-800 was 1.44, indicating successful synthesis of poly(Cr-TPA-4BZ-Py-POP)-800 with a relatively high degree of defects, likely influenced by heteroatom content. XPS analysis [Figure 6(d) and Table S1] verifies the surface composition. The low-resolution spectrum highlights a significant proportion of nitrogen atoms in poly(Cr-TPA-4BZ-Py-POP)-800. Further analysis, including carbon correction using the C=C signal, reveals distinct peaks in the C 1s plot [Figure 6(e)], corresponding to C=C/C–C, C–O/C–N bond, C=O, and COOH. The N 1s spectrum [Figure 6(f)] reveals four peaks representing pyridine-N (N-6), pyrrole-N (N-5), quaternary nitrogen (N-Q), and oxidized-N (N-X). The high proportion of N-6 suggests a successful conversion from N-5, contributing significantly to the CO_2_ adsorption. The O 1s peak [Figure 6(g)] is analyzed, and three peaks correspond to the carbonyl, C–O–C, and C–OH groups; respectively.

**Figure 7:**
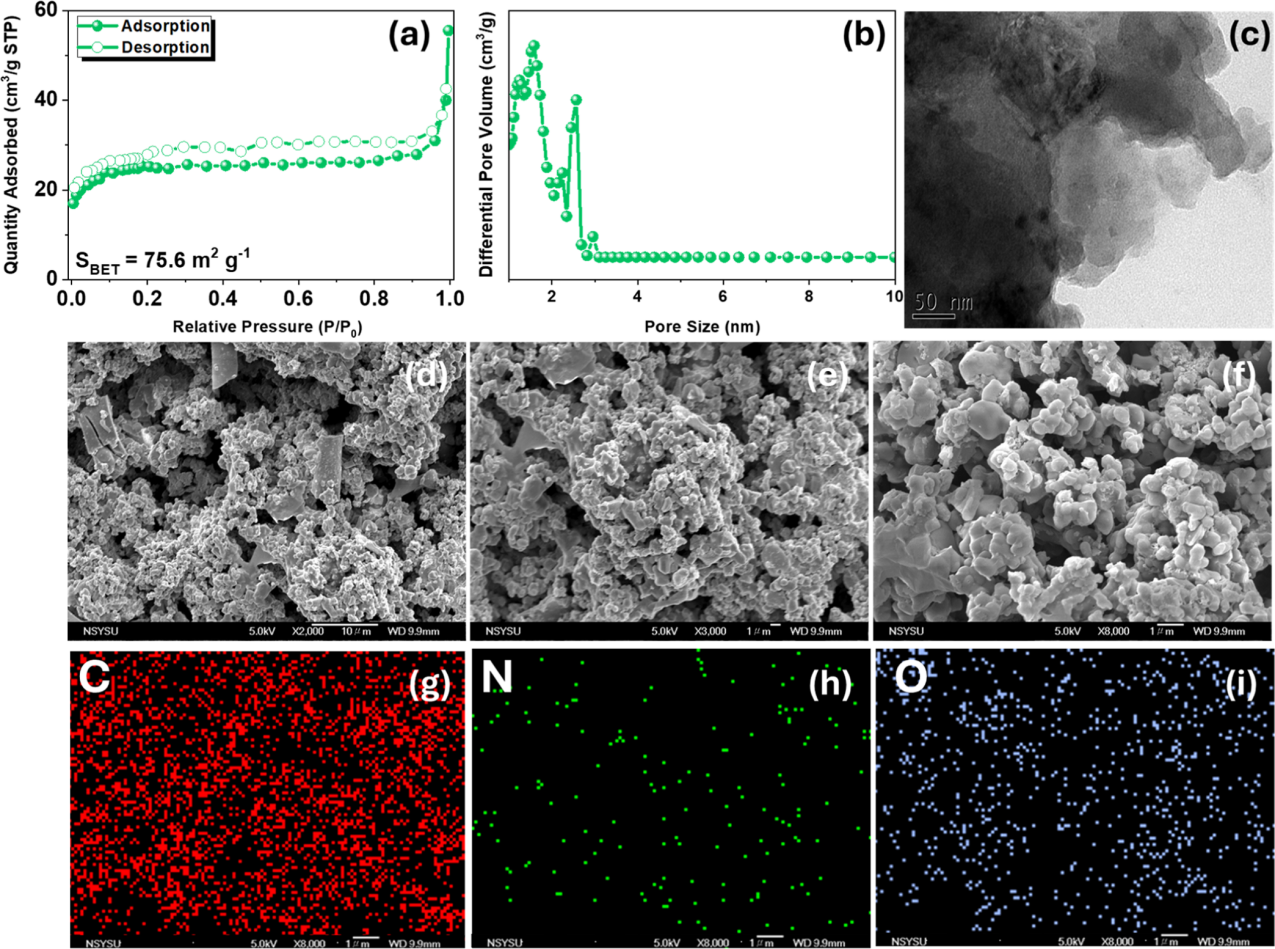
(a, b) BET and pore size profiles, (c) TEM, (d–f) SEM images, and (g–i) EDS-SEM mapping of poly(Cr-TPA-4BZ-Py-POP)-800.

The DFT analysis of Cr-TPA-4BZ-Py-POP [Figure S2] indicates a significant reduction in the energy gap, which is attributed to the highly conjugated nature of pyrene. Specifically focusing on the lowest unoccupied molecular orbital (LUMO) level, it is observed that the LUMO orbital is extensively spread over the pyrene moiety, contributing to its lower energy. Upon breaking the N–O bond within poly(Cr-TPA-4BZ-Py-POP) [Figure S3], the molecule undergoes increased torsion due to single bond rotation, altering its geometry. This change enhances the distinction in the electrostatic potential between N and O atoms compared to the original N–O ring in Cr-TPA-4BZ-Py-POP. Figure 7(a) presents the *S*_BET_ and total pore volume for poly(Cr-TPA-4BZ-Py-POP)-800 N-doped microporous carbon, measuring 75.6 m^2^ g^–1^ and 0.063 cm^3^ g^–1^, respectively. Following IUPAC classification, the isotherms profile of poly(Cr-TPA-4BZ-Py-POP)-800 resembles type I. Utilizing NLDFT, we determined the pore size distribution in the micro-mesoporous range (1.31–2.98 nm) [Figure 7(b)]. The specific surface area significantly increased from 2.2 to 75.6 m^2^ g^–1^, accompanied by increased pore size compared to Cr-TPA-4BZ-Py-POP, enhancing CO_2_ adsorption capabilities and energy storage performance. Figure 7(c) displays HR-TEM images revealing the irregular and disordered morphology of poly(Cr-TPA-4BZ-Py-POP)-800. Figure 7(d–f) displays SEM images highlighting the irregular spherical morphology and disordered arrangement of poly(Cr-TPA-4BZ-Py-POP)-800. The SEM-EDS images of poly(Cr-TPA-4BZ-Py-POP)-800 shown in Figure 7(g–i) reveal a uniform distribution of C, N, and O atoms within poly(Cr-TPA-4BZ-Py-POP)-800. The XPS results for poly(Cr-TPA-4BZ-Py-POP)-800, shown in Figure 6(e,f) and Table S1, indicate a reduced quantity of nitrogen atoms and increased carbon and oxygen atoms within its framework. This suggests a transformation of carbon atoms into a more stable graphitic structure during carbonization and KOH activation [Figure 6(b)].

### CO_2_ Capture of Poly(Cr-TPA-4BZ-Py-POP)-700 and Poly(Cr-TPA-4BZ-Py-POP)-800 N-Doped Microporous Carbon

**Figure 8:**
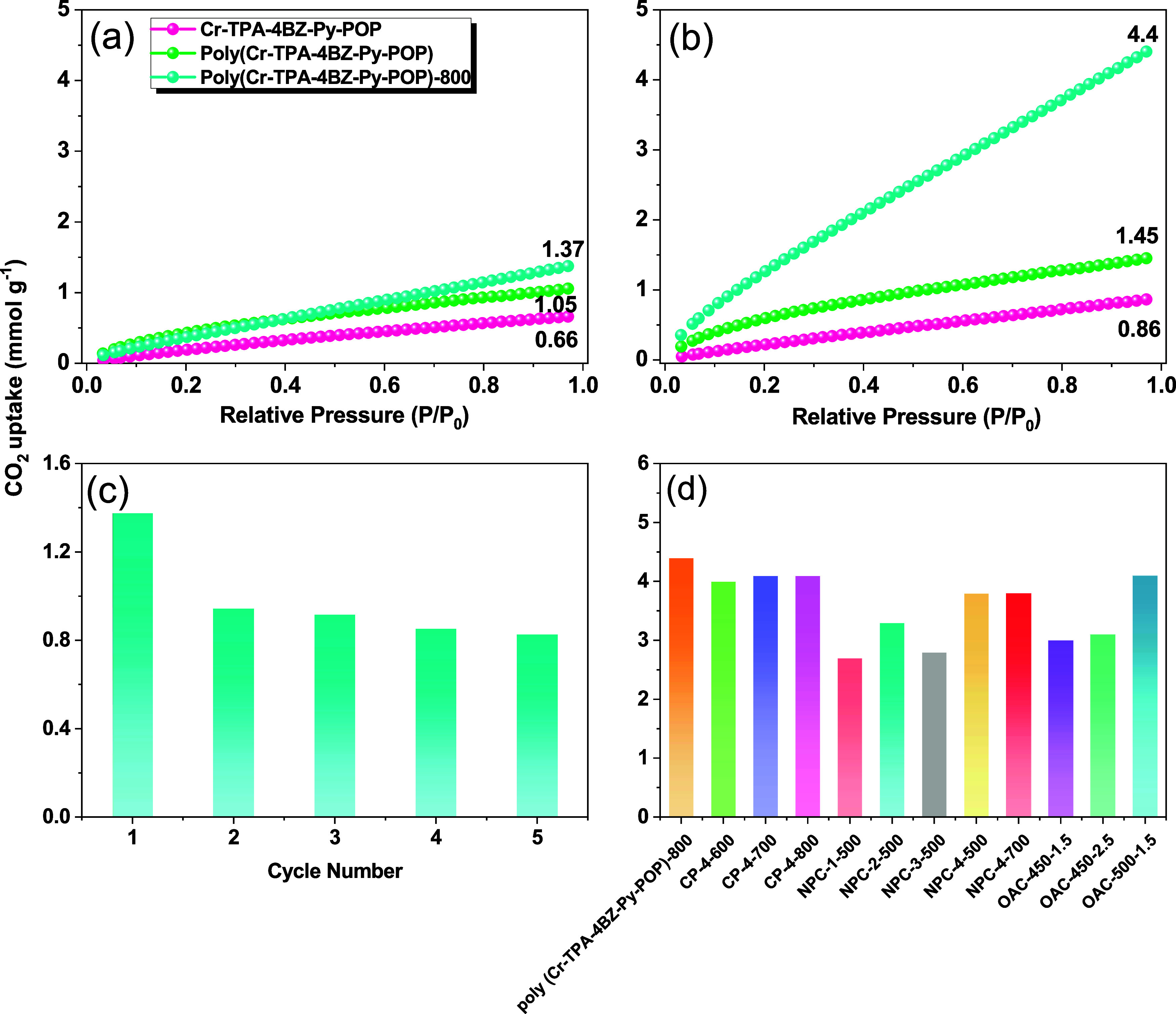
(a, b) CO_2_ uptake of Cr-TPA-4BZ-Py-POP, poly(Cr-TPA-4BZ-Py-POP), and (Cr-TPA-4BZ-Py-POP)-800 under 298 and 273 K, (c) recycling of (Cr-TPA-4BZ-Py-POP)-800 for CO_2_ uptake, and (d) CO_2_ uptake of (Cr-TPA-4BZ-Py-POP)-800 compared with other MCs materials.

In recent research, the conventional emphasis on the high specific surface area of pores for CO_2_ capture has shifted toward recognizing the significance of heteroatoms and microporosity in carbon materials. Carbon materials containing nitrogen and oxygen, such as poly(Cr-TPA-4BZ-Py-POP)-800, have gained popularity due to their successful carbonization and high graphitization. The CO_2_ trapping capacity of Cr-TPA-4BZ-Py-POP, poly(Cr-TPA-4BZ-Py-POP), poly(Cr-TPA-4BZ-Py-POP)-700, and poly(Cr-TPA-4BZ-Py-POP)-800 at different temperatures is illustrated in Figures 8(a,b) and S4. At 298 K, the CO_2_ capture capacities of Cr-TPA-4BZ-Py-POP, poly(Cr-TPA-4BZ-Py-POP), poly(Cr-TPA-4BZ-Py-POP)-700, and poly(Cr-TPA-4BZ-Py-POP)-800 are 0.66, 1.05, 1.17, and 1.37 mmol g^–1^, respectively. These values increase to 0.86, 1.45, 1.87, and 4.40 mmol g^–1^ at 273 K for Cr-TPA-4BZ-Py-POP, poly(Cr-TPA-4BZ-Py-POP), poly(Cr-TPA-4BZ-Py-POP)-700, and poly(Cr-TPA-4BZ-Py-POP)-800, respectively. The remarkable CO_2_ capture capacity of poly(Cr-TPA-4BZ-Py-POP)-800 is attributable to its exceptional porosity, high *S*_BET_, and microporous structure. XPS analysis reveals that incorporating heteroatoms, particularly N-5 and N-6 in the N 1s spectrum, enhances dipole–quadrupole interactions with CO_2_. Additionally, the presence of phenolic OH groups in the O 1s diagram facilitates hydrogen bonding forces [O–H···O=C] and Lewis acid–base interaction (N^+^···=O–C=O) with CO_2_ molecules,^56^ further increasing the affinity of the poly(Cr-TPA-4BZ-Py-POP)-800 for CO_2_ uptake. The possible CO_2_ capture mechanism of poly(Cr-TPA-4BZ-Py-POP)-700 and poly(Cr-TPA-4BZ-Py-POP)-800 can also be seen in Figure S5. CO_2_ molecules are captured by the functionalized pores of poly(Cr-TPA-4BZ-Py-POP)-700 and poly(Cr-TPA-4BZ-Py-POP)-800 through van der Waals forces, which facilitate the attraction between the CO_2_ molecules and the surface of the pores. Additionally, pole–pole interactions between the CO_2_ quadrupole and polar sites within the materials contribute to this adsorption mechanism. The Clausius–Clapeyron equation was employed to calculate the equivalent heat of adsorption (*Q*_st_), with poly(Cr-TPA-4BZ-Py-POP)-800 demonstrating a *Q*_st_ of 48.70 kJ/mol at an adsorption value of about 0.6 mmol/g. This result supports the conclusion that N and −OH groups play a crucial role in CO_2_ trapping. The adsorption cyclicity of poly(Cr-TPA-4BZ-Py-POP)-800 was investigated by repeating adsorption and detachment cycles at 1 bar and 298 K. Figure 8(c) illustrates that after the initial adsorption and detachment (1.37 mmol g^–1^), subsequent cycles exhibit stability of around 0.9 mmol g^–1^. This suggests that after the first adsorption, CO_2_ molecules become trapped between carbon layers, leading to a stable interaction force and demonstrating the material’s high stability in CO_2_ adsorption. Comparative analysis with other nitrogen-doped activated carbon materials (Figure 8(d)) highlights the superior stability and adsorption performance of poly(Cr-TPA-4BZ-Py-POP)-800.^57−59^

### Electrochemical Performance of Cr-TPA-4BZ-Py-POP, Poly(Cr-TPA-4BZ-Py-POP)-700, and Poly(Cr-TPA-4BZ-Py-POP)-800

**Figure 9:**
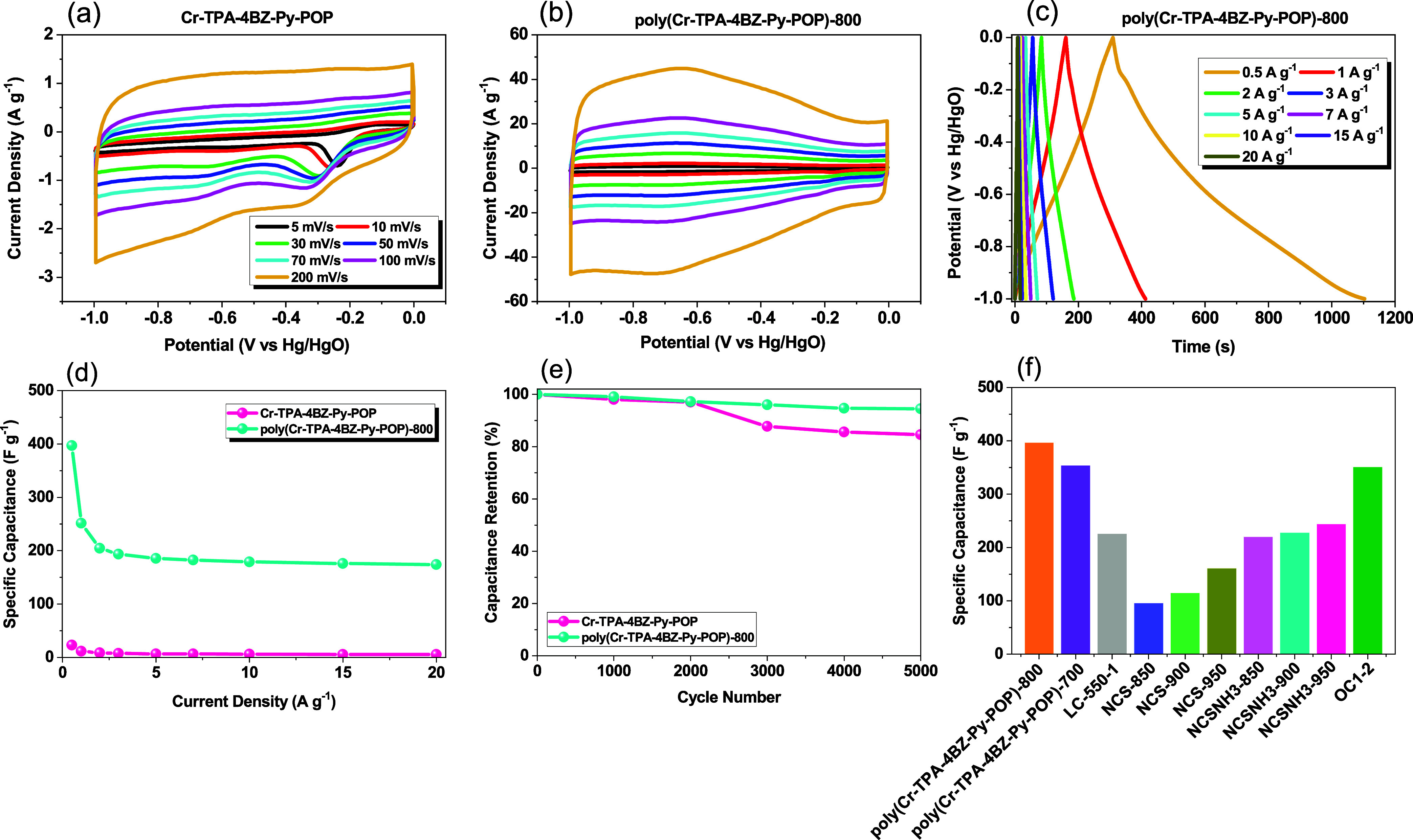
(a, b) CV curves of Cr-TPA-4BZ-Py-POP and poly(Cr-TPA-4BZ-Py-POP)-800, (c) GCD curve of poly(Cr-TPA-4BZ-Py-POP)-800, (d) specific capacitances, (e) 5000 times charge and discharge cycle test of Cr-TPA-4BZ-Py-POP and poly(Cr-TPA-4BZ-Py-POP)-800, and (f) specific capacitance of poly(Cr-TPA-4BZ-Py-POP)-800 compared with other MCs materials.

Recently, heteroatom-containing activated carbon materials have gained prominence in supercapacitor applications due to their cost-effectiveness, high specific surface area, and favorable electrical properties. This study focuses on synthesizing carbon materials with enhanced electrochemical performance by utilizing crown ethers with a high oxygen content and combining them with BZ rings containing N and O atoms. This approach ensures fast electron transfer and optimal pore distribution for improved electrochemical properties. To assess the electrochemical performance, a three-electrode working system was employed. Using 1 M KOH as the electrolyte, glassy carbon, platinum wire, and Hg/HgO served as the working electrode, counter electrode, and reference electrode, respectively. The sample was coated on the working electrode, and cyclic voltammetry (CV) was conducted at 5–200 mV s^–1^ for Cr-TPA-4BZ-Py-POP, poly(Cr-TPA-4BZ-Py-POP)-700, and poly(Cr-TPA-4BZ-Py-POP)-800 within the voltage range of 0 to −1.0 V. The resulting CV curves, as depicted in Figures 9(a,b) and S6(a), exhibit a standard rectangular shape indicative of electric double-layer capacitance (EDLC).^60−66^ Notably, the redox peaks observed in all CV plots are attributed to faradaic redox currents induced by heteroatoms. Given the EDLC behavior and rectangular shape of poly(Cr-TPA-4BZ-Py-POP)-700 and poly(Cr-TPA-4BZ-Py-POP)-800 observed in the galvanostatic charge–discharge (GCD) curves [Figures 9(c) and S6(b)], GCD measurements across a range of 0.5–20 A g^–1^ revealed a capacitance value of 354.41 and 397.2 F g^–1^ at 0.5 A g^–1^ for poly(Cr-TPA-4BZ-Py-POP)-700 and poly(Cr-TPA-4BZ-Py-POP)-800 [Figures 9(d) and S6(c)]. This value, particularly noteworthy after KOH activation, surpasses those of other reported activated carbon materials. Despite a specific surface area of poly(Cr-TPA-4BZ-Py-POP)-800 increase after KOH activation to be 75.6 m^2^ g^–1^ (lower than other reported materials), the superior specific capacitance of poly(Cr-TPA-4BZ-Py-POP)-800 is attributed to the optimal pore distribution and the involvement of heteroatoms, such as nitrogen, in the electrode structure. The complex porous structure of our sample, comprising a combination of micropores and mesopores, underscores the role of micropores in enhancing the electrochemical performance. The contribution of nitrogen atoms, particularly N-5 and N-6, is evident in the redox peaks of the CV curve and the IR drop in the GCD curve. Nitrogen incorporation enhances the electrode conductivity and provides significant pseudocapacitance, leading to further performance improvement. The theoretical specific capacitances of Cr-TPA-4BZ-Py-POP and poly(Cr-TPA-4BZ-Py-POP)-800, as calculated from CV profiles at various scan rates, are summarized in Tables S2 and S3, respectively. Moreover, after 5000 charge–discharge cycles (1 A g^–1^), poly(Cr-TPA-4BZ-Py-POP)-700 and poly(Cr-TPA-4BZ-Py-POP)-800 maintain over 94% of their specific capacitance [Figures 9(e) and S6(d)], highlighting excellent stability despite the involvement of pseudocapacitance in the electrode reaction. The specific capacitance of poly(Cr-TPA-4BZ-Py-POP)-800 was verified by conducting multiple repetitions of GCD measurements across a range of current densities from 0.5 to 20 A g^–1^. These measurements were performed three times to ensure accuracy. Notably, at a current density of 0.5 A g^–1^, the specific capacitance was consistently determined to be 374.69 F g^–1^, as depicted in Figure S7. Figure S8 shows that the Coulombic efficiency of Cr-TPA-4BZ-Py-POP and poly(Cr-TPA-4BZ-Py-POP)-800 was determined to be 90.1 and 99.81%, respectively. Comparison with other nitrogen-doped activated carbon materials is presented in Figure 9(f), further affirming the superior electrochemical performance of poly(Cr-TPA-4BZ-Py-POP)-700 and poly(Cr-TPA-4BZ-Py-POP)-800.^67−70^

It is necessary to compute the percentages of surface-controlled capacitive and diffusion-controlled processes to the total charge obtained (*Q*_total_) to appropriately implement the charging harvesting mechanism. Regarding the Trasatti attempt, in this case, the stored charge is the outer charge (*Q*_outer_), and only surface operations took place when the applied potential sweep rate reached its maximum value. Figure S9(a,b) show the relationship between capacity *Q* and (*v*)^−0.5^, where (*v*) is the possible scan rate. The intercept of the relationship between *Q* and (*v*)^−0.5^ can be used to compute the value of (*Q*_outer_) in accordance with eq 1.
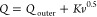
1

On the other hand, the total charge (*Q*_total_), which can be found by plotting 1/*Q* against (*v*)^0.5^, as shown in Figure S9(c,d), is the stored charge in this scenario when the potential scan rate reaches its minimum values that provide ample time for the diffusion of the ions. In accordance with eq 2.
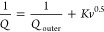
2

As a result, the yields of diffusion-controlled faradic of Cr-TPA-4BZ-Py-POP and poly(Cr-TPA-4BZ-Py-POP)-800 are 91.8 and 21.5%, respectively (Figure S9(e,f)). Figure S10(a) displays Nyquist plots obtained from electrochemical impedance spectroscopy (EIS) conducted on our Cr-TPA-4BZ-Py-POP and poly(Cr-TPA-4BZ-Py-POP)-800. The accompanying fitted electrical circuit includes important elements such as series resistance (*R*_s_), charge transfer resistance (*R*_ct_), and constant phase elements representing electric double-layer capacitance (CPE–EDL) and pseudocapacitance (CPE–P), along with a Warburg element (*Z*_w_), as depicted in Figure S10(b). The ohmic resistances for Cr-TPA-4BZ-Py-POP and poly(Cr-TPA-4BZ-Py-POP)-800 were measured at 27.12 and 23.66 Ω, respectively, before and after curing reactions. Furthermore, the charge transfer resistances were found to be 34 096 and 14 283 Ω for Cr-TPA-4BZ-Py-POP and poly(Cr-TPA-4BZ-Py-POP)-800, respectively. Figure S10(c) presents the frequency-dependent magnitude Bode plots for these Cr-TPA-4BZ-Py-POP and poly(Cr-TPA-4BZ-Py-POP)-800 electrodes. These plots exhibit slanted lines with a negative slope at low frequencies, indicating lower resistance, and at high frequencies, confirming the impressive capacitive characteristics of these electrode materials. Figure S10(d) illustrates the frequency-dependent phase angles for the Cr-TPA-4BZ-Py-POP and poly(Cr-TPA-4BZ-Py-POP)-800 electrodes. The knee frequencies, identified at a phase angle of −45°, signify the point where the resistive and capacitive properties of the electrode become equivalent. This knee frequency serves as a well-established indicator of a compound’s rate capacity. Cr-TPA-4BZ-Py-POP and poly(Cr-TPA-4BZ-Py-POP)-800 demonstrate knee frequencies of 51.03 and 250.60 Hz, respectively. We utilized a CR2032 coin cell configuration, comprising an anode, cathode, separator, bottom and top covers, and electrolyte, to construct a symmetric supercapacitor. The cathode and anode were fabricated by using our poly(Cr-TPA-4BZ-Py-POP)-800 material. Electrochemical experiments were conducted within a potential range of −0.3 to 0.2 V, employing a range of scan rates from 5 to 200 mV s^–1^. Figure S11(a) displays the CV profile of a symmetric coin cell of poly(Cr-TPA-4BZ-Py-POP)-800, obtained across scan rates ranging from 5 to 200 mV s^–1^. The near-rectangular shape observed in the CV curve is attributed to the combined effects of the EDLC and the pseudocapacitive nature of the poly(Cr-TPA-4BZ-Py-POP)-800. The increased current density with escalating scan speeds, as illustrated in Figure S11(a), is evidence of the improved kinetics and stability of these electrode materials. Additionally, the graphical representation of GCD curves for poly(Cr-TPA-4BZ-Py-POP)-800 at diverse current densities, Figure S11(b), reveals triangular forms with slight bends, indicative of both EDLC properties and pseudocapacity. This is shown in Figure S11(c); poly(Cr-TPA-4BZ-Py-POP)-800 has a capacitance of 159.2 F g^–1^ at 1 A g^–1^, along with a power density of 500 W/kg and an energy density of 5.53 Wh kg^–1^ [Figure S11(a)]. Table S4 outlines the electrochemical performance of the poly(Cr-TPA-4BZ-Py-POP)-800 coin cell in comparison with other supercapacitor electrodes derived from PBZ-based carbon materials. Figure S12(a) depicts the correlation between capacity *Q* and (*v*)^−0.5^, where (*v*) represents the scan rate. The intercept of this relationship between *Q* and (*v*)^−0.5^ can be utilized to calculate the value of (*Q*_outer_) following the procedure specified in eq 1. Conversely, the total charge (*Q*_total_), determined by plotting 1/*Q* against (*v*)^0.5^, as illustrated in Figure S12(b), represents the stored charge in this context when the potential scan rate reaches its lowest values, allowing sufficient time for ion diffusion. This calculation follows the prescribed procedure in eq 2. Consequently, the diffusion-controlled faradic yield of the poly(Cr-TPA-4BZ-Py-POP)-800 coin cell is 38%. In the case of the coin cell (a two-electrode system), as the scan rate rises, the surface capacitive contribution also increases. Figure S12(c) demonstrates that at a scan rate of 200 mV s^–1^, the surface capacitive contribution and diffusion control contribution were determined to be 94.3% and 5.66%, respectively. We conducted EIS measurements on a coin cell utilizing poly(Cr-TPA-4BZ-Py-POP)-800 as the electrode, simulating a real supercapacitor device. The EIS spectra were effectively modeled using an equivalent electric circuit (refer to Figures S13(a,b)), underscoring the potential of the poly(Cr-TPA-4BZ-Py-POP)-800-based device for practical energy storage applications. Additionally, Figure S13(c) depicts frequency-dependent magnitude Bode plots for the poly(Cr-TPA-4BZ-Py-POP)-800 electrode, revealing a characteristic pattern: slanted lines with a negative slope at lower frequencies and reduced resistance at higher frequencies, indicating remarkable capacitive properties. Furthermore, Figure S13(d) illustrates the frequency-dependent phase angles for the poly(Cr-TPA-4BZ-Py-POP)-800 electrodes. The knee frequencies observed at a phase angle of −45° (at 590 Hz) signify the point where resistive and capacitive traits of the electrode are balanced. This knee frequency serves as a reliable indicator of the compound’s rate capacity.

## Conclusions

To conclude, a unique class of POPs [Cr-TPA-4BZ-Py-POP] was developed through the combination of Cr-TPA-4BZ-Br_4_ and Py-T through the Sonogashira reaction. The conventional three-step process for producing the Cr-TPA-4BZ-Br_4_ monomer involves the synthesis of Schiff bases, reduction, and a Mannich condensation reaction. Furthermore, poly(Cr-TPA-4BZ-Py-POP)-800 N-doped microporous carbon was synthesized through the carbonization and chemical activation of poly(Cr-TPA-4BZ-Py-POP), with a notable carbon dioxide adsorption capacity of 4.4 mmol g^–1^ at 273 K for poly(Cr-TPA-4BZ-Py-POP)-800. In electrochemical terms, poly(Cr-TPA-4BZ-Py-POP)-800 demonstrates a high specific capacitance value of 397.2 F g^–1^ with a high capacitance retention (94%), suggesting its potential as a robust supercapacitor. The remarkable properties are primarily attributed to the doping heteroatom ratio, resulting in the structural arrangement. In today’s challenging environmental conditions, the outstanding characteristics of poly(Cr-TPA-4BZ-Py-POP)-800 N-doped microporous carbon indicate significant potential for addressing both environmental and energy-related challenges.

## Supporting Information Available

Details about the experimental and characterization methods and electrochemical analysis; schematic scheme of the synthesized compounds; spectroscopic data; DFT of Cr-TPA-4BZ-Py-POP and poly(Cr-TPA-4BZ-Py-POP); XRD profile of poly(Cr-TPA-4BZ-Py-POP)-800; and electrochemical and EIS data of poly(Cr-TPA-4BZ-Py-POP)-700 and poly(Cr-TPA-4BZ-Py-POP)-800 (PDF)
