# Supplementary material for: Robust Nitrogen-Doped Microporous Carbon via Crown Ether-Functionalized Benzoxazine-Linked Porous Organic Polymers for Enhanced CO2 Adsorption and Supercapacitor Applications
Source: ACS Appl Mater Interfaces. 2024 Jul 22;16(31):40858–72. doi: 10.1021/acsami.4c05645 (PMC11311139; doi:10.1021/acsami.4c05645)
Supplement: Supplementary file 1 — am4c05645_si_001.pdf [file am4c05645_si_001.pdf]

Supporting Information

Robust Nitrogen-Doped Microporous Carbon via Crown Ether Functionalized Benzoxazine Linked Porous Organic Polymers for Enhanced CO_2 Adsorption and Supercapacitor Applications

Mohamed Gamal Mohamed[a,b]*, Bo-Xuan Su[a] and Shiao-Wei Kuo[a,c]*

[a]Department of Materials and Optoelectronic Science, Center of Crystal Research, National Sun Yat-Sen University, Kaohsiung 804, Taiwan.

[b]Chemistry Department, Faculty of Science, Assiut University, Assiut 71516, Egypt.

[c]Department of Medicinal and Applied Chemistry, Kaohsiung Medical University, Kaohsiung 807, Taiwan.

Corresponding authors:
E-mail: mgamal.eldin12@yahoo.com (M. G. Mohamed) and kuosw@faculty.nsysu.edu.tw (S. W. Kuo).

Characterization

FTIR spectra were collected on a Bruker Tensor 27 FTIR spectrophotometer with a resolution of 4 cm^{-1} by using KBr disk method. ^{13}C nuclear magnetic resonance (NMR) spectra were examined by using an INOVA 500 instrument with DMSO as the solvent and TMS as the external standard. Chemical shifts are reported in parts per million (ppm). The thermal stabilities of the samples were performed by using a TG Q-50 thermogravimetric analyzer under a N_2 atmosphere; the cured sample (ca. 5 mg) was put in a Pt cell with heating rate of 20 °C min^{-1} from 100 to 800 °C under a N_2 flow rate of 60 mL min^{-1}. Solid-state ^{13}C NMR was measured by JEOL JNM-LA300 spectrometer and standard CPMAS probe at 75.577 MHz. Wide-angle X-ray diffraction (WAXD) patterns were measured by the wiggler beamline BL17A1 of the National Synchrotron Radiation Research Center (NSRRC), Taiwan. A triangular bent Si (111) single crystal was used to get a monochromated beam having a wavelength (λ) of 1.33 Å. The morphologies of the polymer samples were examined by Field emission scanning electron microscopy (FE-SEM; JEOL JSM7610F) and also by transmission electron microscope (TEM) using a JEOL-2100 instrument at an accelerating voltage of 200 kV. X-ray Photoelectron Spectroscopy (XPS): XPS was measured on a X-ray Photoelectron Spectrometer System (Thermo Scientific). The X-ray monochromator used micro-focused Al-Kα radiation. BET surface area and porosimetry measurements of samples (ca. 40–100 mg) were measured using BEL Master^{TM}/BEL sim^{TM} (v. 3.0.0). N_2 adsorption and desorption isotherms were generated through incremental exposure to ultrahigh-purity N_2 (up to ca. 1 atm) in a liquid N_2 (77 K) bath. Surface parameters were calculated using BET adsorption models in the instrument's software. The prepared samples' pore size was determined using nonlocal density functional theory (NLDFT).

Synthesis of Dibenzo[18]crown [Cr][1]

In a microwave container, catechol (2.20 g, 20.0 mmol), NaOH (2.24 g, 40.0 mmol) and bis(2-chloroethyl) ether (2.86 g, 20.0 mmol) were dissolved in anhydrous DMSO (15 mL) and stirred at 70 °C for 4 h. The mixture was then poured into a mixture of ice water (300 mL) and HCl (37 wt%, 10 mL) and stirred for 2 h at 25 °C. The resulting solution was extracted three times with CH_2Cl_2. The combined $CH2Cl_2$ phases were dried ($MgSO_4$) and filtered. The solvent was evaporated using a rotary evaporator and then the solid powder was washed with acetone (100 mL). The solid was filtered off and dried under vacuum (50 °C, 24 h) to afford dibenzo[18]crown-6 as fibrous needles (1.50 g, 69%); m.p. 162.5–163.5 °C (by DSC). FTIR (KBr): 3051 (aromatic C–H stretching), 2922 (C–H stretching), 1255 (C–O–C stretching). ^{1}H NMR (DMSO-d_6, 25 °C, 500 MHz): δ = 6.94 (d, 4H), 6.85 (d, 4H), 4.06-3.87 (m, 8H), 3.86-3.71 (m, 8H). ^{13}C NMR (DMSO-d_6, 25 °C, 125 MHz): δ = 139.40, 129.26, 124.45, 113.07, 112.32.

Synthesis of trans-Di(nitrobenzo)[18]crown-6 (Dibenzo-crownether-2NO_2) [Cr-2NO_2][1]

A solution of dibenzo[18]crown-6 (3.00 g, 9.23 mmol) in a mixture of $CHCl_3$ (80 mL) and acetic acid (10 mL) in a 250-mL two-neck flask was stirred at room temperature for 1 h. A mixture of acetic acid (5 mL) and HNO_3 (2 mL) was added dropwise and then the mixture was heated at 50 °C for 24 h. The white solid was filtered off and washed with MeOH to afford the title compound (2.50 g, 83%); m.p. 251–252 °C (by DSC). FTIR (KBr): 3051 (aromatic C–H stretching), 1517, 1346 (NO_2 stretching). 1H NMR (DMSO-d_6, 25 °C, 500 MHz): δ = 7.90 (s, 2H), 7.88 (s, 2H), 7.17 (s, 2H), 4.06-3.87 (m, 8H), 3.86-3.71 (m, 8H).13C NMR (DMSO-d_6, 25 °C, 125 MHz): δ = 153.77, 147.68, 140.56, 117.62, 111.27, 106.58, 68.44, 68.03.

Synthesis of trans-Di(aminobenzo)[18]crown-6 (Dibenzo-crownether-2NH_2) [Cr-2NH_2][1]

Dibenzo-crownether-2NO_2 (5.00 g, 11.1 mmol) and Pd/C (0.050 g, 0.47 mmol) were placed in a mixture of dry 1,4-dioxane (40 mL) and absolute EtOH (20 mL) in a 250-mL two-neck flask and then heated under reflux for 1 h at 100 °C under a N_2 atmosphere. $NH_2NH_2 \cdot H_2O$ (10 mL) was added slowly into the mixture, which was then heated at 100 °C for 2 days. The mixture was filtered to remove unreacted Pd/C and then the solvent was evaporated under reduced pressure to afford a white powder (3.50 g, 70%); m.p. 205–207 °C (by DSC). FTIR (KBr): 3428 and 3356 (N–H stretching). ^{1}H NMR (DMSO-d_6, 25 °C, 500 MHz): δ = 6.63 (s, 2H), 6.24 (s, 2H), 6.06 (s, 2H), 4.63 (s, 4H), 4.06-3.87 (m, 8H), 3.86-3.71 (m, 8H).^{13}C NMR (DMSO-d_6, 25 °C, 125 MHz): δ = 149.22, 143.51, 139.22, 115.68, 105.35, 100.73, 69.44, 67.64.

Synthesis of Dibenzo-crownether-4NO_2 [Cr-TPA-4NO_2][2]

The mixture of Cr-2NH_2 (1 g, 2.56 mmol), 1-fluoro-4-nitrobenzene (1.63 mL, 15.38 mmol), K_2CO_3 (6.37 g, 46.09 mmol), Cu (0.033 g, 0.52 mmol) and DMF (30 mL) was heated and stirred under N_2 at 110 °C for 24 hours. Following the reaction, the mixture underwent filtration to remove the solid, and subsequently, the DMF solvent was evaporated under reduced pressure, resulting in the formation of a viscous brown solid. The brown solid was then dissolved in the DCM and added dropwise into the methanol to obtain Cr-TPA-4NO_2 as the brown powder.

Synthesis of Dibenzo-crownether-4NO_2 [Cr-TPA-4NH_2][2]

The mixture of Cr-TPA-4NO_2 (1 g, 1.14 mmol), Pd/C (1.63 mL, 6.86 mmol), DO (40 mL), and EtOH (20 mL) was heated and stirred under N_2 at 90 °C for 1 hour. After one hour, a gentle addition of 1.89 mL (38.96 mmol) of $NH_2NH_2.H_2O$ was made to the mixture, which was subsequently heated at 90 °C for two days. The resulting mixture was subjected to filtration to eliminate any unreacted Pd/C, followed by evaporation of the solvent under reduced pressure. This process led to the formation of Cr-TPA-4NH_2 as a white powder.

The experimental details for DFT calculations

Density functional theory (DFT) calculations were conducted at the B3LYP/6-31G(d) level using the Gaussian 09W program. The consideration of the D3BJ dispersion correction was essential to address long-range and non-covalent interactions effectively. Moreover, the global minimum of each conformer in the ground-state geometry was identified through harmonic vibrational frequency analysis to determine the lowest energy conformer for subsequent analysis. The calculations encompassed the determination of the highest occupied molecular orbital (HOMO), lowest unoccupied molecular orbital (LUMO), and molecular electrostatic potential (MESP) at optimized geometries utilizing the same level of theory.

Electrochemical Analysis

Working Electrode Cleaning: Before using, the glassy carbon electrode (GCE) was polished several times with 0.05-µm alumina powder, washed with EtOH after each polishing step, cleaned through sonication (5 min) in a water bath, washed with EtOH, and then dried in the oven at 50 ^{o}C.

Electrochemical Characterization: The electrochemical experiments were performed in a three-electrode cell using an Autolab potentiostat (PGSTAT204) and 1 M KOH as the aqueous electrolyte. The GCE was used as the working electrode (diameter: 5.61 mm; 0.2475 cm^2); a Pt wire was used as the counter electrode; Hg/HgO (RE-1B, BAS) was the reference electrode. All reported potentials refer to the Hg/HgO potential. A slurry was prepared by dispersing Cr-TPA-4BZ-Py-POP or poly(Cr-TPA-4BZ-Py-POP)-800 (2 mg), carbon black (2 mg), and Nafion (10 wt%) in a mixture of (EtOH/ H_2O) (200 µL: 800 µL) and then sonicating for 1 h. A portion of this

slurry (10 µL) was pipetted onto the tip of the electrode, which was then dried in air for 30 min before use. The electrochemical performance was studied through CV at various sweep rates (5–200 mV s^{-1}) and through the GCD method in the potential range from 0 to -1.00 V (vs. Hg/HgO) at various current densities (0.5–20 A g^{-1}) in 1 M KOH as the aqueous electrolyte solution.

The specific capacitance was calculated from the GCD data using the equation.

$$C_s = (I\Delta t)/(m\Delta V)$$

Where C_s (F g^{-1}) is the specific capacitance of the supercapacitor, I (A) is the discharge current, ΔV (V) is the potential window, Δt (s) is the discharge time, and m (g) is the mass of the NPC on the electrode. The energy density (E, W h kg^{-1}) and power density (P, W kg^{-1}) were calculated using the equations.

$$E = 1000C(\Delta V)^2/(2 \times 3600)$$

$$P = E/(t/3600)$$

Electrochemical Analysis in Two-Electrode Symmetric Supercapacitor System

The slurry prepared by mixing poly(Cr-TPA-4BZ-Py-POP)-800, carbon black, and Nafion (10 wt. %) was coated onto a flexible Kuraray carbon paper (0.1 mm in thickness) with an effective area of 1 cm × 1 cm and then dried at 100 °C overnight in a vacuum oven. The mass loading of active material on the current collector was 0.8 mg cm^{-2}. The two working electrodes were separated with filter paper and infiltrated with potassium hydroxide (1 M) aqueous solution.

The specific capacitance was calculated from galvanostatic charge-discharge experiments using the following equation:

$$Cs = 2 \text{ x } (I\Delta t)/(m\Delta V)$$

Where Cs (F/g) is specific capacitance of the supercapacitor, I (A) is the discharge current, ΔV (V) is the potential window, Δt (s) is the time, and m (g) is the mass of porous carbon on the one electrode. The energy density (E, Wh kg^{-1}) and power density (P, W kg^{-1}) were calculated using the equations.

$$E = C(\Delta V)^2/7.2$$

$$P = E/(t/3600)$$

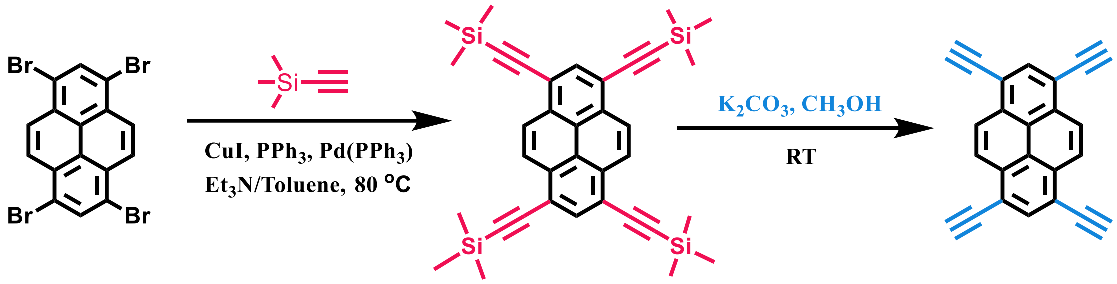

Scheme S1. Synthesis of Py-T.

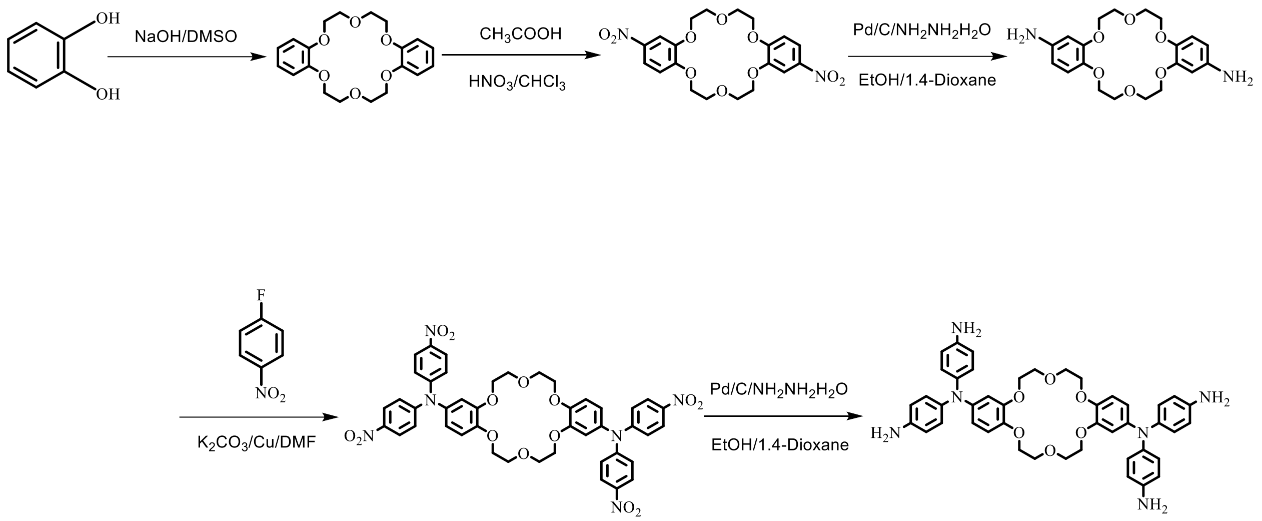

Scheme S2. Synthesis of Cr-TPA-4NH_2.

Table S1. The XPS fitted data of poly(Cr-TPA-4BZ-Py-POP)-800.

**Percentage of total elements(%)**		
**C**	N	O
**73.5**	8.9	17.6

**C-Area fraction (%)**			
**C=C/C-C**	C-O/C-N	C=O	COOH
**75.74**	10.12	6.56	7.58

**N-Area fraction (%)**			
**N-6**	N-5	N-Q	N-X
**60.00**	29.93	8.65	1.42

**O-Area fraction (%)**		
**C=O**	C-O-C	C-OH
**51.57**	41.62	6.80

Table S2. The specific capacitance of Cr-TPA-4BZ-Py-POP from CV profiles at different scan rates (three-electrode system).

Scan Rate ($mV\ s^{-1}$)	Specific capacitance ($F\ g^{-1}$)
**5**	**44.1**
**10**	**22.9**
**30**	**11.7**
**50**	**9.4**
**70**	**8.5**
**100**	**7.9**
**200**	**6.7**

Table S3. The specific capacitance of poly(Cr-TPA-4BZ-Py-POP)-800 from CV profiles at different scan rates (three-electrode system).

Scan Rate ($mV\ s^{-1}$)	Specific capacitance ($F\ g^{-1}$)
**5**	**197.1**
**10**	**189.9**
**30**	**179.9**
**50**	**177.2**
**70**	**176.1**
**100**	**173.9**
**200**	**170.4**

Table S4. Comparison of electrochemical properties of poly(Cr-TPA-4BZ-Py-POP)-800 with other benzoxazine-based carbon materials.

Porous Carbon Materials	E (Wh kg^{-1})	Ref.
CNM-800	11.2 (45 W kg^{-1})	3
NOPC-bis-CN-3	10.8 (45 W kg^{-1})	4
NOSHPCs	5.67 (275 W kg^{-1})	5
PCCGNs	3.37 (250 W kg^{-1})	6
Poly(RES-HP-BZ)-800	16.63 (346 W kg^{-1})	7
Melamine Foam	4.33 (250 W kg^{-1})	8
Phenolic resin	7.3 (50 W kg^{-1})	9
**poly(Cr-TPA-4BZ-Py-POP)-800**	**5.53 (500 W kg^{-1})**	This work

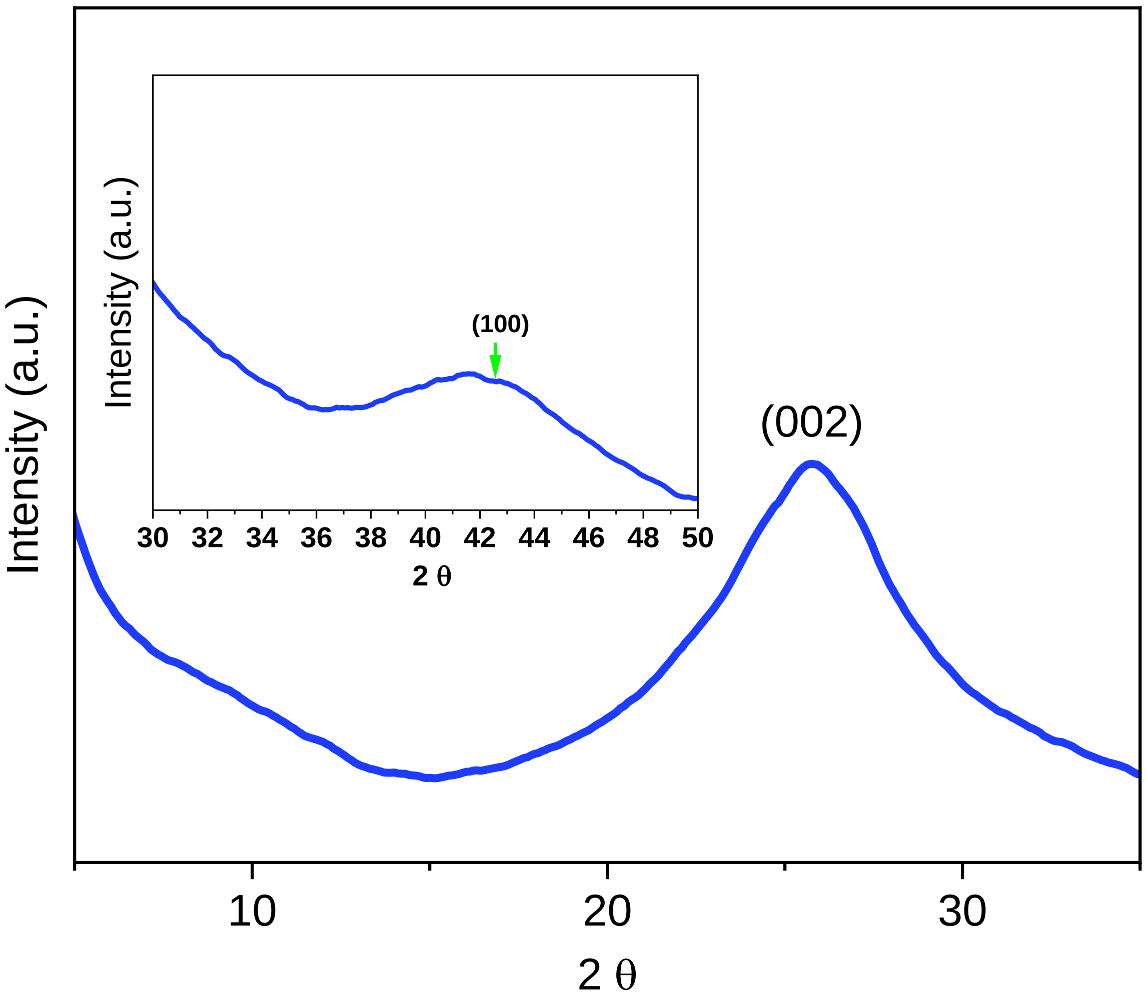

Figure S1. XRD profile of poly(Cr-TPA-4BZ-Py-POP)-800.

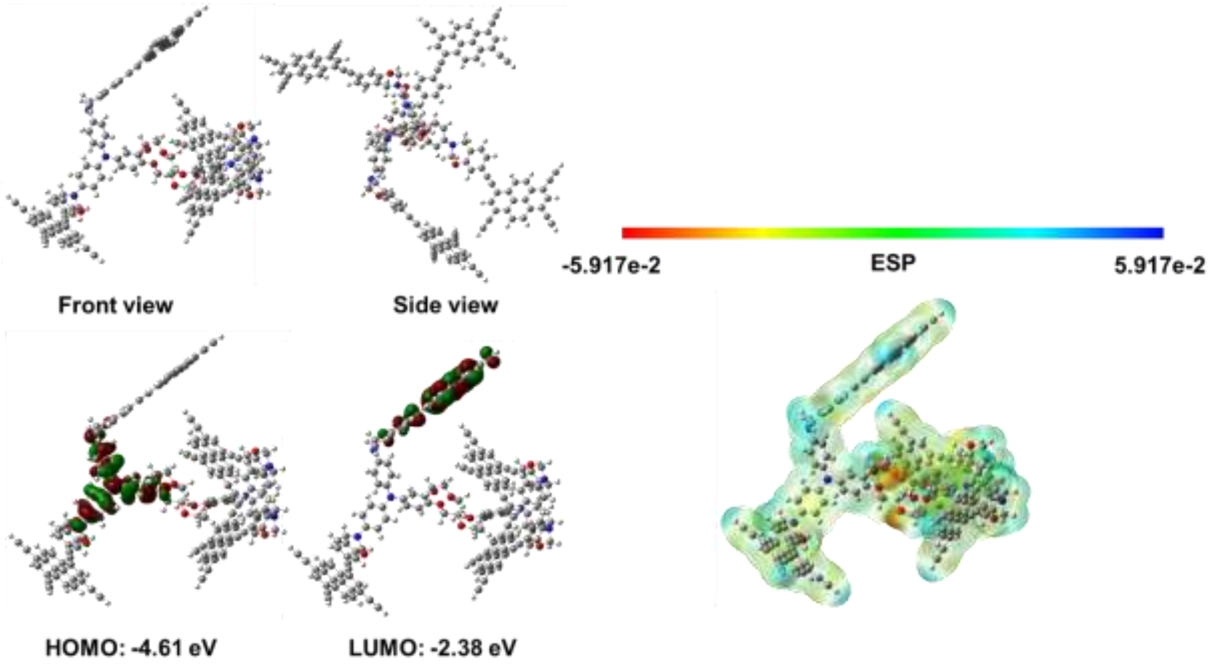

Figure S2. DFT of Cr-TPA-4BZ-Py-POP.

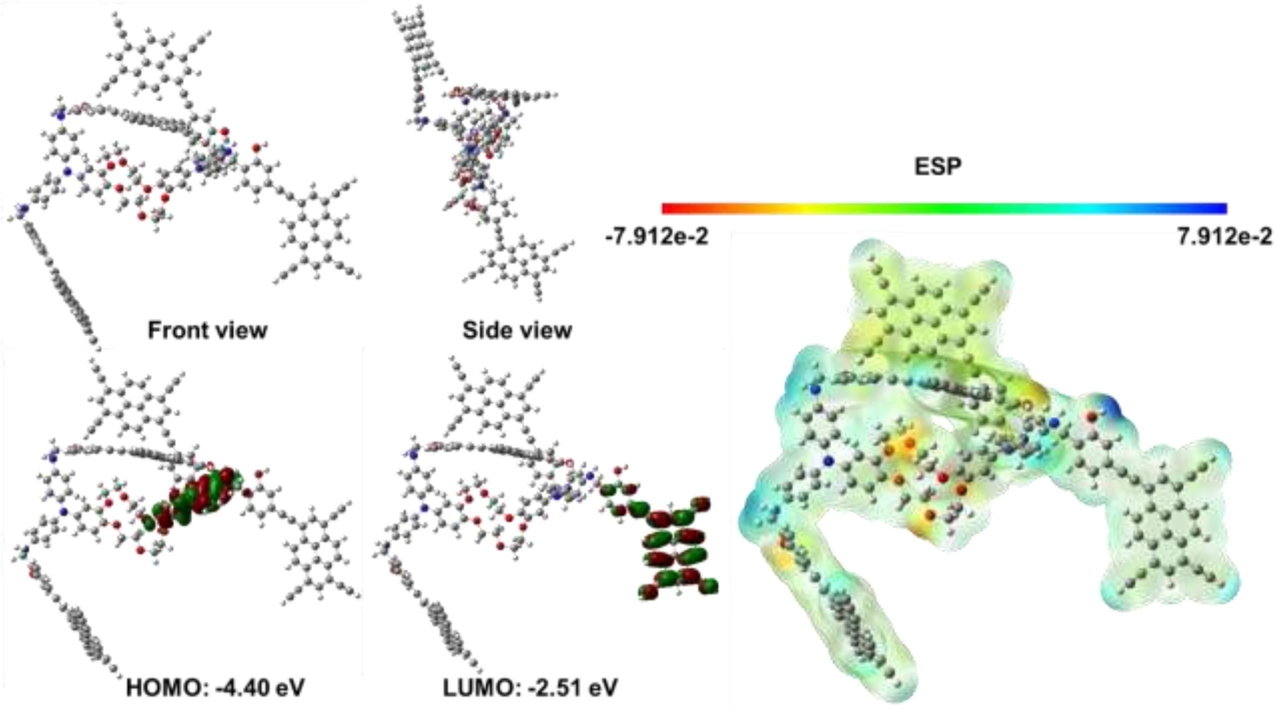

Figure S3. DFT of poly(Cr-TPA-4BZ-Py-POP).

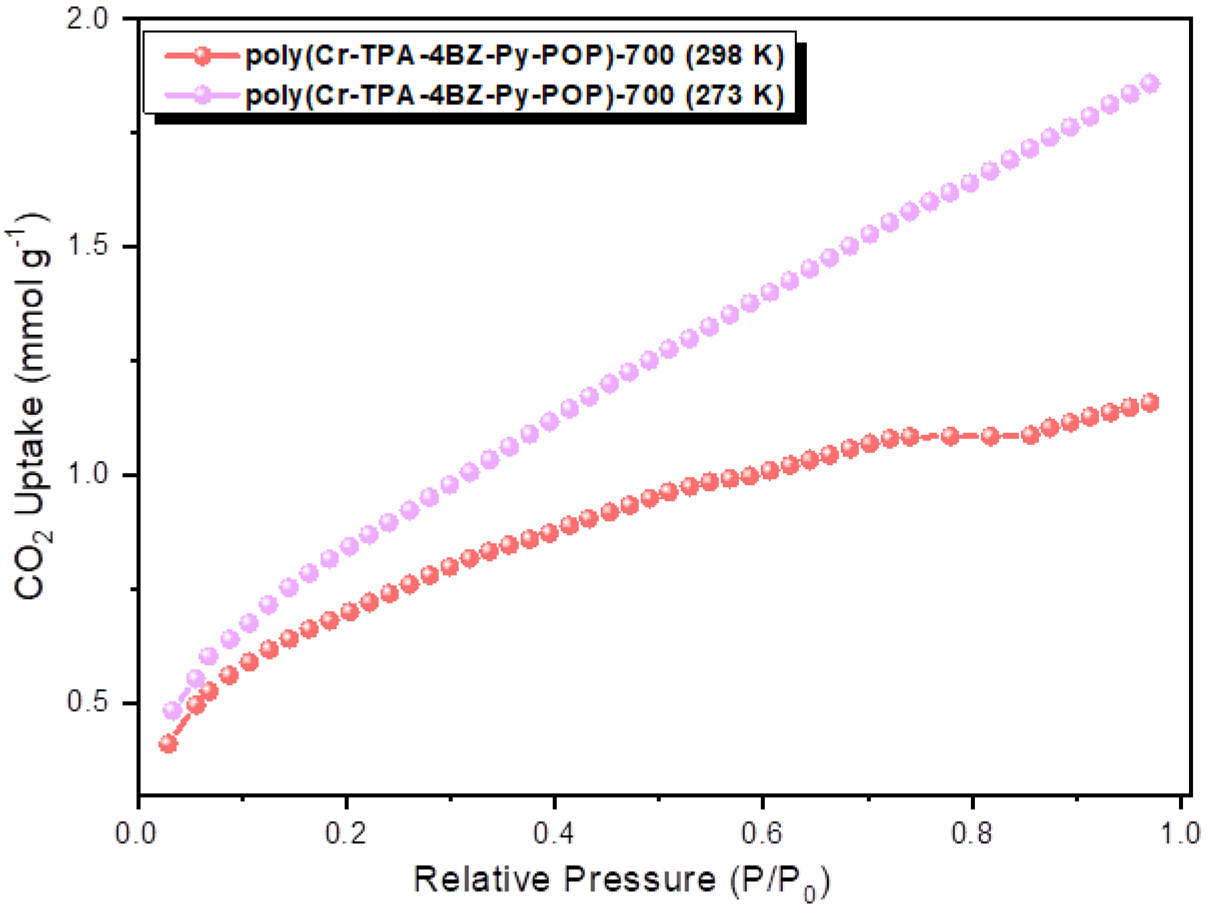

Figure S4. CO_2 uptake of poly(Cr-TPA-4BZ-Py-POP)-700 at 298 and 273 K.

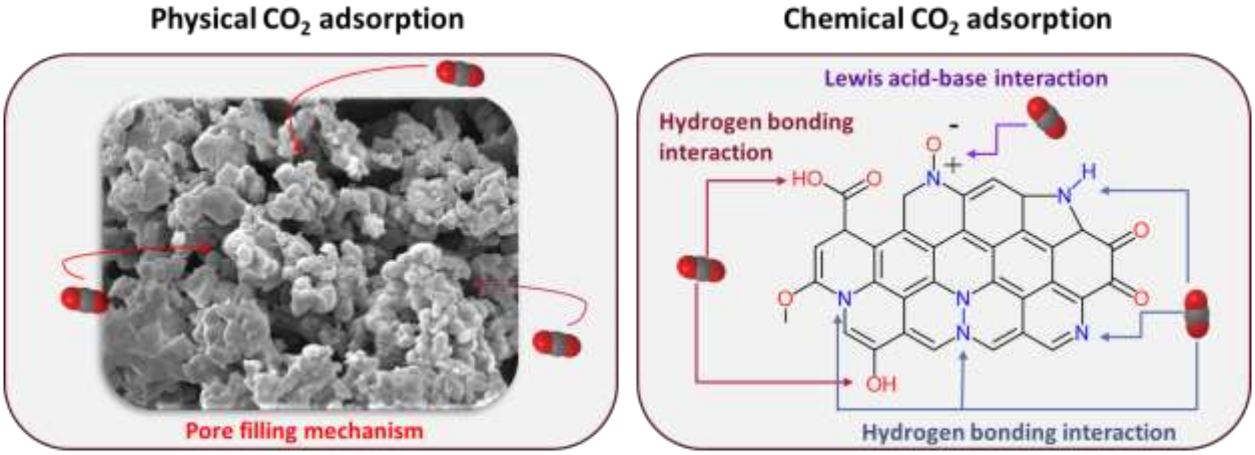

Figure S5. The possible CO_2 capture mechanism of poly(Cr-TPA-4BZ-Py-POP)-700 and poly(Cr-TPA-4BZ-Py-POP)-800.

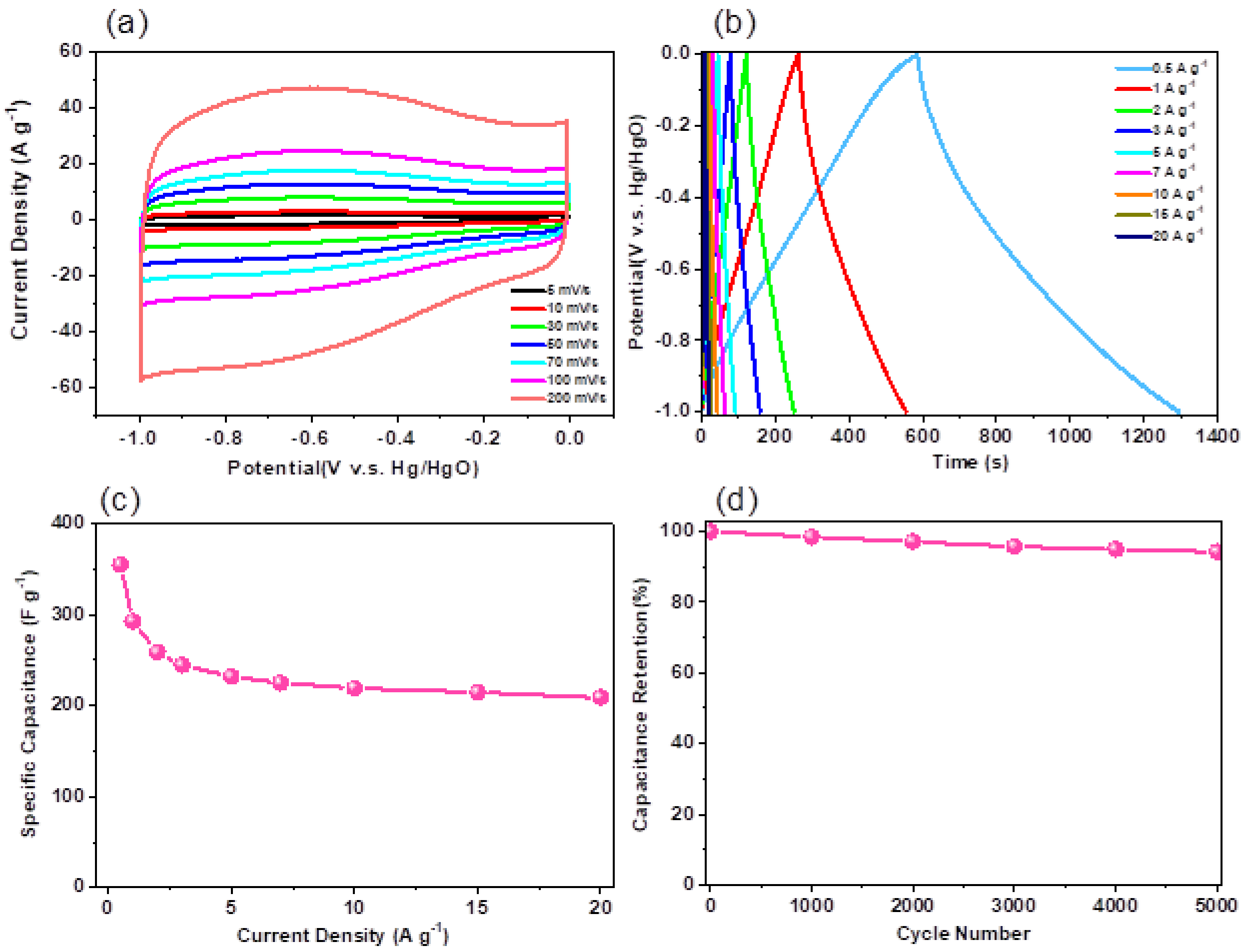

Figure S6. (a) CV, (b) GCD, (c) specific capacitance, and (d) capacitance retention of poly(Cr-TPA-4BZ-Py-POP)-700 based on a three-electrode system.

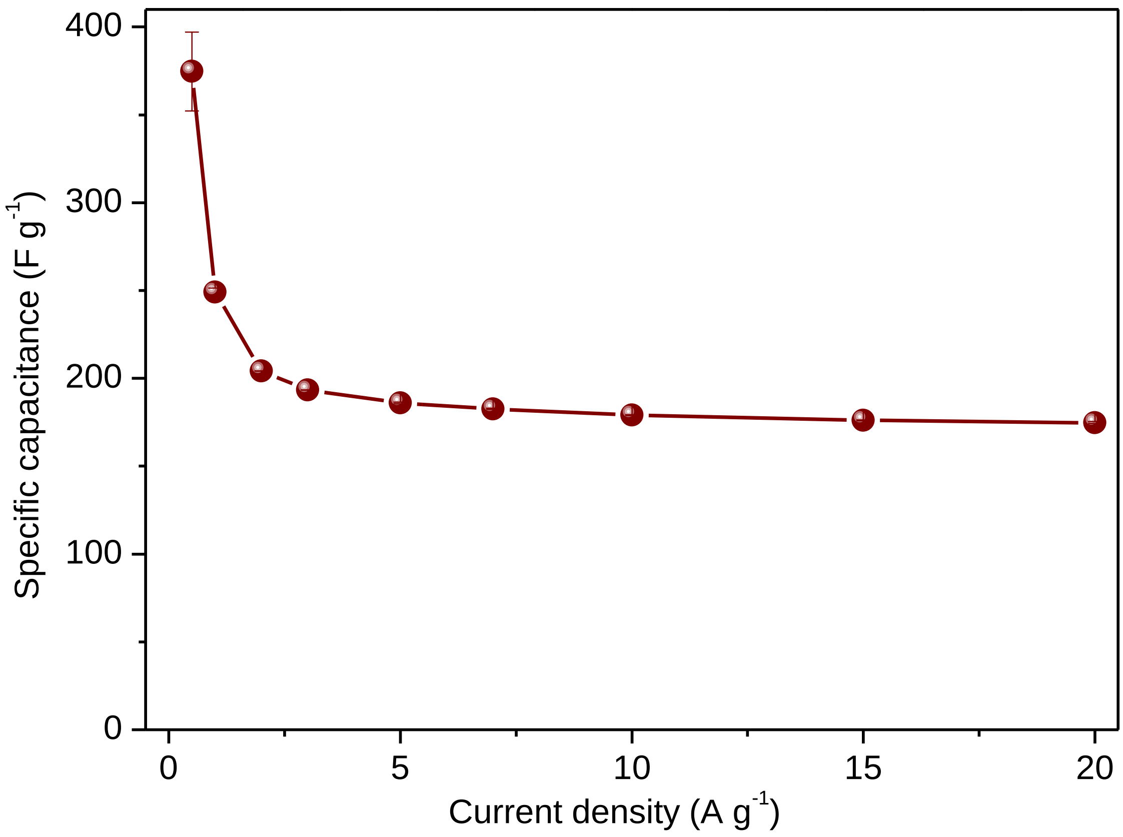

Figure S7. Error bar of poly(Cr-TPA-4BZ-Py-POP)-800 derived from GCD measurements.

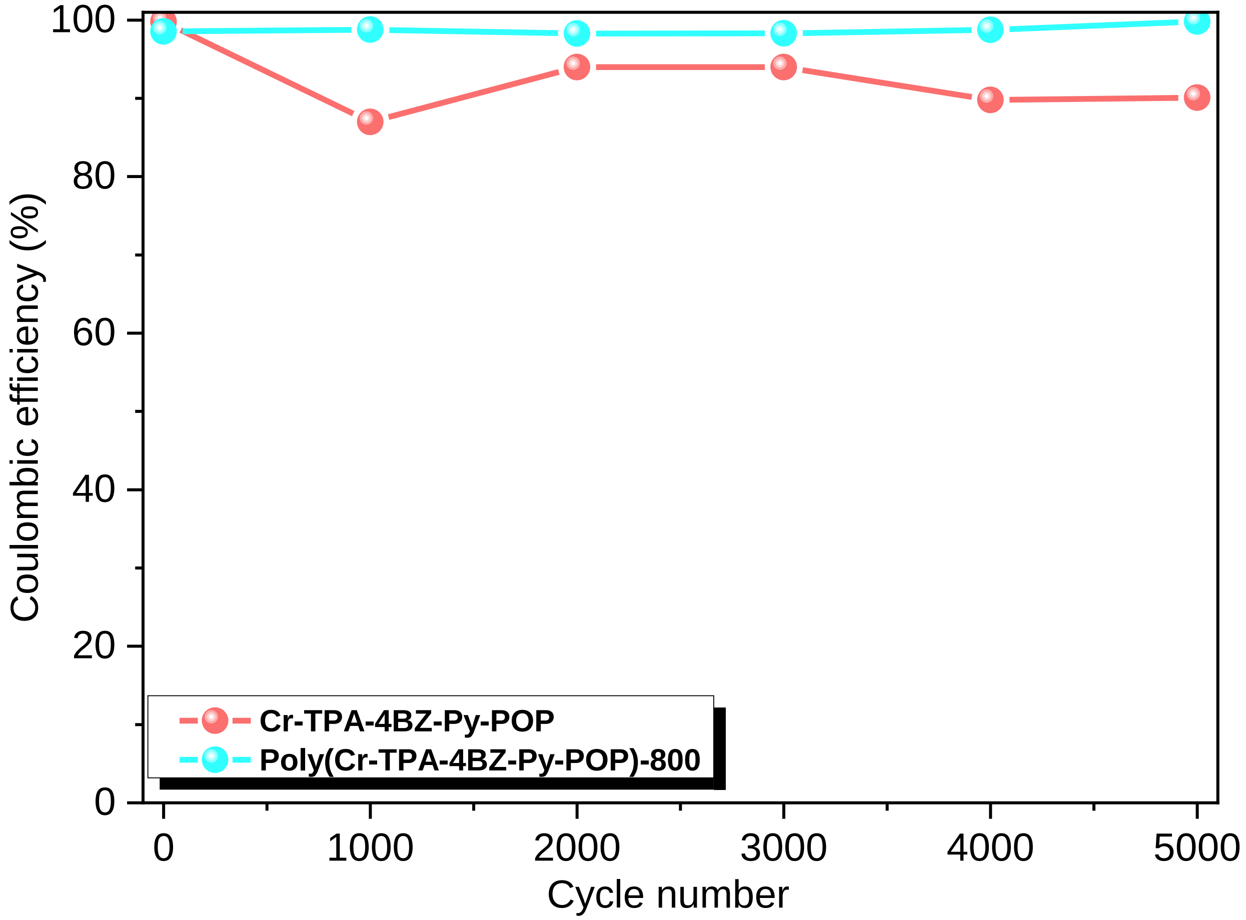

Figure S8. Coulombic efficiency of Cr-TPA-4BZ-Py-POP and poly(Cr-TPA-4BZ-Py-POP)-800.

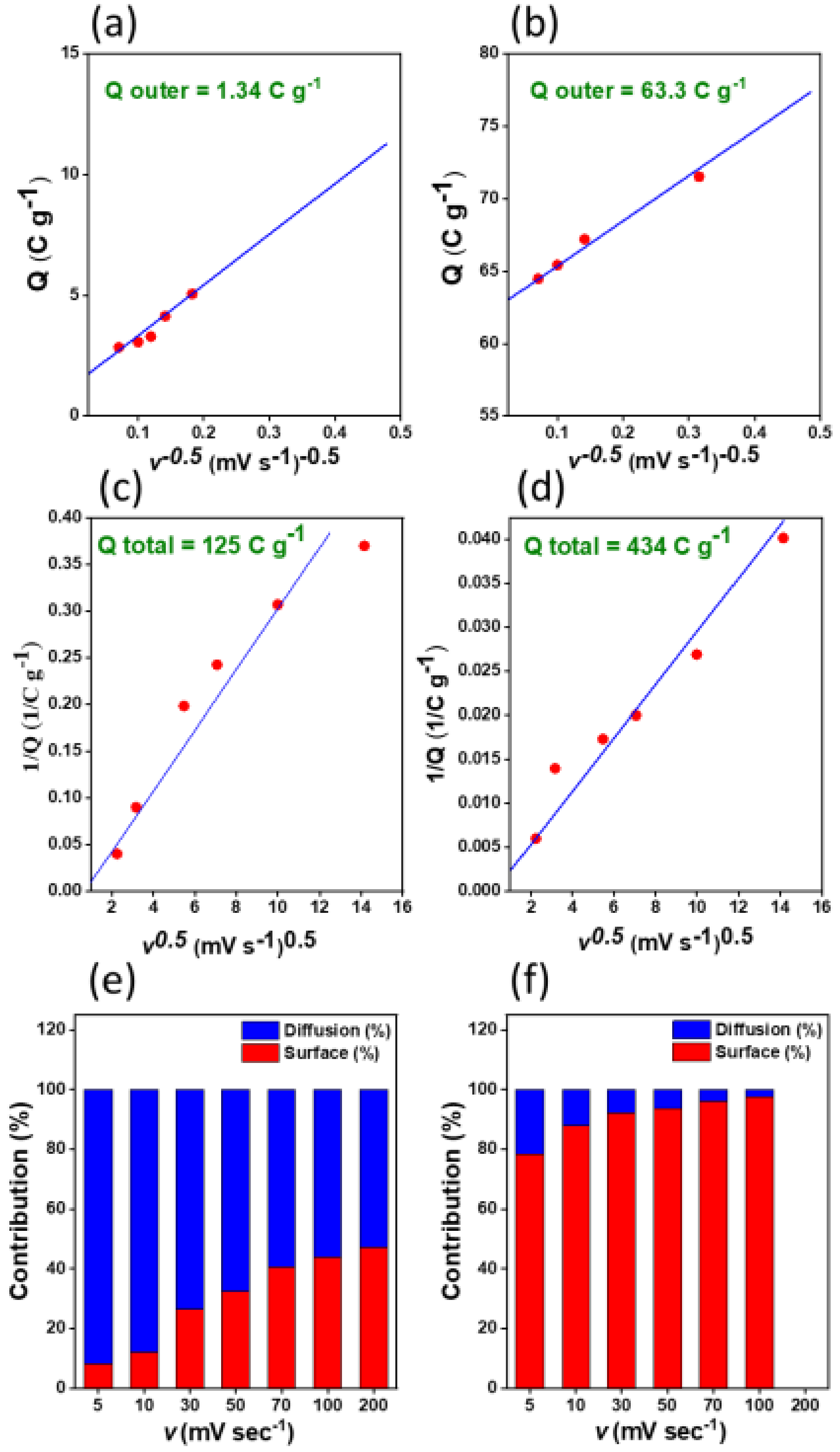

Figure S9. (a and b) Relation between Q (C g^{-1}) and $v^{-0.5}$ (mV s^{-1})$^{-0.5}$, (c and d) 1/Q vs. $v^{0.5}$, and (e and f) surface contribution and diffusion-contribution as a percentage for (a, c, e) Cr-TPA-4BZ-Py-POP, and (b, d, f) poly(Cr-TPA-4BZ-Py-POP)-800, respectively.

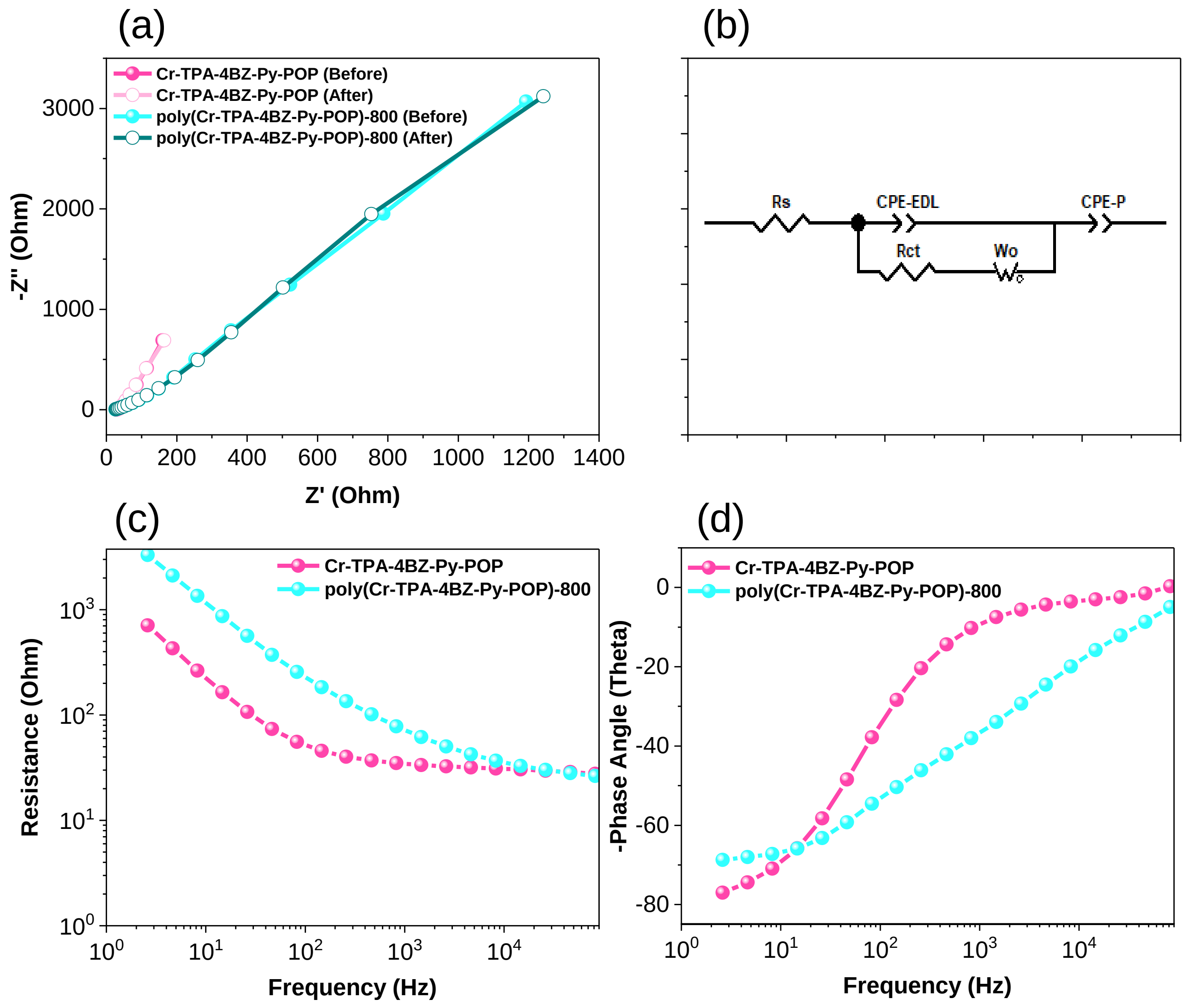

Figure S10. Nyquist plots of (a) Cr-TPA-4BZ-Py-POP and poly(Cr-TPA-4BZ-Py-POP)-800, before and after fitting; (b) Equivalent electric circuit used for fitting (c) Bode plots of frequency-dependent resistance (magnitude), and (d) Bode plots of frequency-dependent phase angles for Cr-TPA-4BZ-Py-POP and poly(Cr-TPA-4BZ-Py-POP)-800.

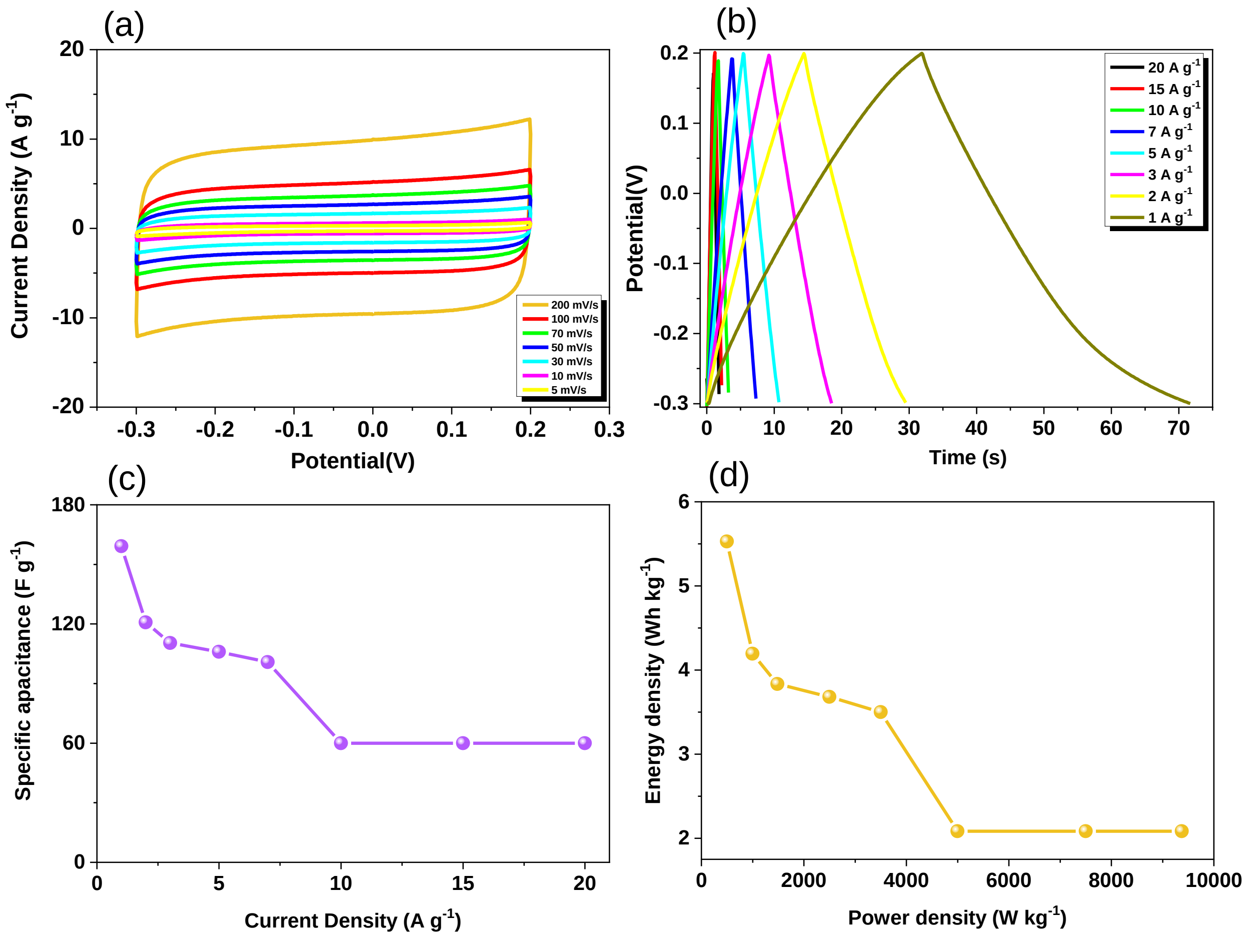

Figure S11. (a) CV, (b) GCD, (c) specific capacitance, and (d) Ragone plot of poly(Cr-TPA-4BZ-Py-POP)-800 based on a symmetric coin cell.

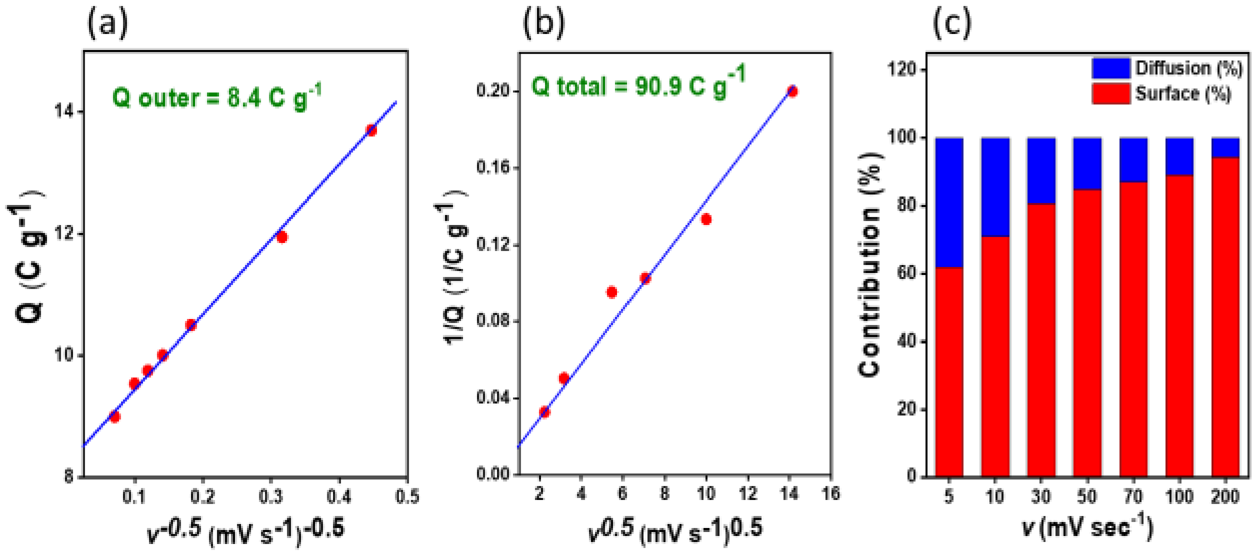

Figure S12. (a) Relation between Q (C g^{-1}) and $v^{-0.5}$ (mV s^{-1})$^{-0.5}$, (b) 1/Q vs. $v^{0.5}$, and (c) surface contribution and diffusion-contribution as a percentage for poly(Cr-TPA-4BZ-Py-POP)-800.

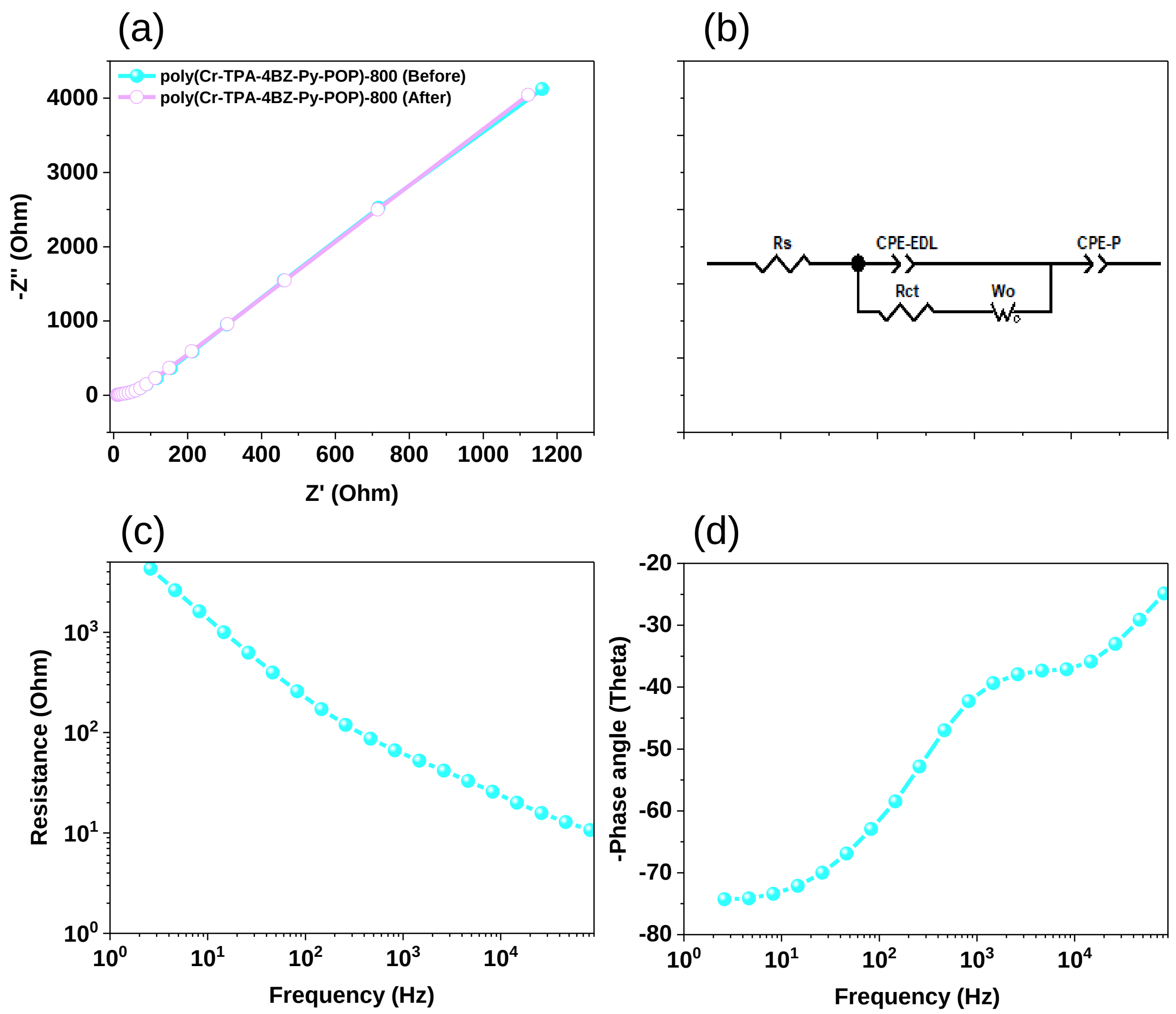

Figure S13. Nyquist plots of (a) poly(Cr-TPA-4BZ-Py-POP)-800 (device), before and after fitting; (b) Equivalent electric circuit used for fitting (c) Bode plots of frequency-dependent resistance (magnitude), and (d) Bode plots of frequency-dependent phase angles for poly(Cr-TPA-4BZ-Py-POP)-800.

References

[1] Mohamed, M. G.; Kuo, S. W. Crown Ether-Functionalized Polybenzoxazine for Metal Ion Adsorption. *Macromolecules* **2020**, *53*, 2420-2429, DOI: 10.1021/acs.macromol.9b02519.

[2] Mohamed, M. G.; Chang, W. C.; Chaganti, S. V.; Sharma, S. U.; Lee, J. T.; Kuo, S. W. Dispersion of ultrastable crown-ether-functionalized triphenylamine and pyrene-linked porous organic conjugated polymers with single-walled carbon nanotubes as high-performance electrodes for supercapacitors. *Polym. Chem.* **2023**, *14*, 4589-4601, DOI: 10.1039/D3PY00708A.

[3] Wang, L.; Sun, J. P.; Zhang, H. H.; Xu, L.; Liu, G. J. Preparation of benzoxazine-based N-doped mesoporous carbon material and its electrochemical behaviour as supercapacitor. *J. Electroanal. Chem.* **2020**, *868*, 114196. DOI: 10.1016/j.jelechem.2020.114196.

[4] Liu, Y.; Cao, L.; Luo, J.; Peng, Y.; Ji, Q.; Dai, J.; Zhu, J.; Liu, X. Biobased nitrogen-and oxygen-codoped carbon materials for high-performance supercapacitor. *ACS Sustain. Chem. Eng.* **2018**, *7*, 2763-2773. DOI: 10.1021/acssuschemeng.8b05947.

[5] Jiang, J.; Wang, M.; Zhao, W.; Liu, H.; Wang, Y.; Song, P.; Wang, Z. Hierarchical porous carbon materials derived from N, O, S-Containing Bio-Based polybenzoxazine for Supercapacitors. *Eur. Polym. J.* **2023**, *191*, 112054. DOI: 10.1016/j.eurpolymj.2023.112054.

[6] Jiang, J.; Wang, M.; Zhao, W.; Cao, Y.; Shi, R.; Wang, Z. Potassium acetate activation strategy for synthesizing multiple heteroatom-doped porous carbons containing 2D nanosheets from polybenzoxazine for supercapacitor applications. *Eur. Polym. J.* **2024**, *208*, 112856. DOI: 10.1016/j.eurpolymj.2024.112856.

[7] Ejaz, M.; Mohamed, M. G.; Chen, Y. T.; Zhang, K.; Kuo, S. W. Porous carbon materials augmented with heteroatoms derived from hyperbranched biobased benzoxazine resins for enhanced CO_2 adsorption and exceptional supercapacitor performance. *J. Energy Storage* **2024**,

78, 110166. DOI: 10.1016/j.est.2023.110166.

[8] Zhang, R.; Jing, X. X.; Chu, Y. T.; Wang, L.; Kang, W. J.; Wei, D. H.; Li, H. B.; Xiong, S. L. Nitrogen/oxygen co-doped monolithic carbon electrodes derived from melamine foam for high-performance supercapacitors. *J. Mater. Chem. A* **2018**, *6*, 17730–17739. DOI: 10.1039/C8TA06471G.

[9] Zhou, J.; Qiu, Z. P.; Zhou, J. K.; Si, W. J.; Cui, H. Y.; Zhuo, S. P. Hierarchical porous carbons from alkaline poplar bark extractive-based phenolic resins for supercapacitors. *Electrochim. Acta* **2015**, *180*, 1007–1013. doi.org/10.1016/j.electacta.2015.09.038.
